# The contour effect: Differences in the aesthetic preference and stress response to photo-realistic living environments

**DOI:** 10.3389/fpsyg.2022.933344

**Published:** 2022-12-01

**Authors:** Nour Tawil, Leonie Ascone, Simone Kühn

**Affiliations:** ^1^Lise Meitner Group for Environmental Neuroscience, Max Planck Institute for Human Development, Berlin, Germany; ^2^Max Planck Dahlem Campus of Cognition, Berlin, Germany; ^3^International Max Planck Research School on the Life Course (LIFE), Berlin, Germany; ^4^Clinic and Policlinic for Psychiatry and Psychotherapy, University Medical Center Hamburg- Eppendorf, Hamburg, Germany; ^5^Max Planck UCL Centre for Computational Psychiatry and Ageing Research, Berlin, Germany

**Keywords:** interior design, contours, style, aesthetic preference, stress response, sex-related differences, biophilia

## Abstract

The interest in the response to contours has recently re-emerged, with various studies suggesting a universal preference for curved over angular stimuli. Although no consensus has yet been reached on the reasons for this preference, similar effects have been proposed in interior environments. However, the scarcely available research primarily depends on schematic or unmatched stimuli and faces heterogeneity in the reported results. In a within-subject design, we investigated the claimed contour effect in photo-realistic indoor environments using stimulus material previously tested in virtual reality (VR). A total of 198 online participants rated 20 living room images, exclusively manipulated on the contours (angular vs. curved) and style (modern vs. classic) levels. The scales represented aesthetic (beauty and liking) and stress (rest and stress) responses. Beyond our main focus on contours, we additionally examined style and sex effects to account for potential interactions. Results revealed a significant main effect of contours on both aesthetic (η^2^_g_ = 1–2%) and stress (η^2^_g_ = 8–12%) ratings. As expected, images of curved (vs. angular) contours scored higher on beauty, liking, and rest scales, and lower on stress. Regarding interactions with style, curvature was aesthetically preferred over angularity only within images depicting modern interiors, however, its positive effect on stress responses remained significant irrespective of style. Furthermore, we observed sex differences in aesthetic but not in stress evaluations, with curvature preference only found in participants who indicated female as their sex. In sum, our study primarily confirms positive effects of curvature, however, with multiple layers. First, the impact on aesthetic preference seems to be influenced by individual and contextual factors. Second, in terms of stress responses, which might be especially relevant for designs intended to promote mental-health, the consistent effects suggest a more generalizable, potentially biophilic characteristic of curves. To the best of our knowledge, this is the first study to demonstrate these effects in fully-matched, photo-realistic, and multi-perspective interior design stimuli. From the background of a previous VR trial from our research group, whereby the same rooms did not elicit any differences, our findings propose that static vs. immersive presentations might yield different results in the response to contours.

## Introduction

The human-environment interaction has recently been in the focus of many fields of research from humanities to natural sciences, with bourgeoning interdisciplinary efforts attempting to link characteristics of sensory stimuli to psychological responses and mental states. It is now widely accepted that the aesthetics of our physical surroundings, whether natural or man-made, can play a meaningful role in shaping our mood and overall well-being (Wohlwill, 1976; Gibson, 1979; Evans and McCoy, 1998; Gifford, 2002; Evans, 2003; Staats et al., 2003; Elliot and Maier, 2014; Coburn et al., 2020). Yet, little is known about this relationship within built settings, particularly with regards to the identification of features that drive the observed effects and the underlying psychological mechanisms (Eberhard, 2009; Graham et al., 2015; Coburn et al., 2017; Bower et al., 2019).

### Affect: Aesthetic preference vs. stress response

During an aesthetic experience, visual properties and higher-order content are segregated along multiple brain regions involved in the regulation of reward and judgment (Chatterjee and Vartanian, 2014). An active simultaneous involvement of emotional, cognitive, and contextual factors is suggested to mediate such aesthetic encounters (Chatterjee and Vartanian, 2014; Coburn et al., 2017). Among the various proposed theoretical models addressing the alternating roles of affect and cognition, it has been commonly agreed that evaluations/judgments are the result of bottom-up stimulus properties and top-down appraisals (Leder et al., 2004; Mastandrea and Bartoli, 2011; Chatterjee and Vartanian, 2016; Chamberlain, 2022). Experiencing a positive and pleasant aesthetic encounter will therefore increase positive affect (Leder et al., 2004), potentially benefiting health and well-being (Coburn et al., 2017). Despite the remaining open questions of which subjective (top-down) and objective (bottom-up) features exactly drive (interindividual) differences in empirical aesthetics, consistent response patterns were found and attributed to certain aesthetic primitives. Stimulus properties such as contour shape (Bar and Neta, 2007; Vartanian et al., 2013), color (Palmer et al., 2013; Strauss et al., 2013; Elliot and Maier, 2014), as well as symmetry (Tyler, 2003; Bertamini et al., 2018, 2019), order, complexity (Nadal et al., 2010; Van Geert and Wagemans, 2021), and global image properties (e.g., fractality) were proposed as objective predictors of aesthetic preference (Chamberlain, 2022). However, other approaches stress the idiosyncrasies of preferences, demonstrating a stronger shared taste for natural or naturally inspired aesthetic domains as opposed to artifacts of human culture (Vessel et al., 2018).

Although aesthetic preference has been long argued as part of the affective domain (within the broad pleasantness dimension), a differentiation between conscious responses (i.e., preference as cognitive accompaniments of an emotion) and innate ones (i.e., affects) has been made (Ulrich, 1983). Beyond preference, physical environments can affect the stress response inducing changes on the psychological, physiological (bodily), hormonal (cortisol), and behavioral levels (Ulrich et al., 2008). For instance, it has become increasingly clear that the exposure to natural environments can reduce psychological and physiological stress (Ulrich et al., 1991; Berto, 2014), with new evidence of a causal effect on stress-related brain regions (Sudimac et al., 2022). Such mechanisms are linked to the biophilia hypothesis which suggests an innate evolutionary-based tendency for humans to connect with nature (Wilson, 1984). This hypothesis has been extended onto man-made environments, and frameworks of biophilic design have emerged (Browning et al., 2014; Kellert and Calabrese, 2015; Salingaros, 2015, 2019; Coburn et al., 2019), proposing that elements such as light, colors, fractals, representation of nature, and also curves, not only increase perceived aesthetic value, but can also reduce stress in humans (Salingaros, 2019; Yin et al., 2020).

### The contour effect, learnt or innate?

Among the many environmental features, contour shapes have been proposed to play a fundamental role in how we perceive our surroundings (Loffler, 2008; Chuquichambi et al., 2022). Over the last two decades, the investigation of contours has recently regained momentum with seemingly robust evidence supporting a universal positive effect of curvature (Bar and Neta, 2007; Gómez-Puerto et al., 2016; Palumbo and Bertamini, 2016; Cotter et al., 2017). When presented with images showing lines, abstract/ geometric shapes, drawings/images of real objects, or sketches/ images of products (e.g., packages, car interiors), it appears that people prefer curved over angular or edgy stimuli (Gordon, 1909; Leder and Carbon, 2005; Bar and Neta, 2006; Silvia and Barona, 2009; Westerman et al., 2012; Palumbo et al., 2015; Chuquichambi et al., 2021). Findings were replicated under different experimental paradigms, further exploring other possible stimulus-related mediators, but also interindividual differences (moderators) of this phenomenal effect (refer to Tawil et al., 2021 and Corradi and Munar, 2019 for a more detailed review).

However, the origin of this phenomenon is still under debate, with no consensus reached as to the psychological mechanisms that drive it. On the one hand, the cumulative evidence from humans of different ages (including newborns and infants) (Fantz and Miranda, 1975; Jadva et al., 2010) and cultures (Gómez-Puerto et al., 2018), as well as non-human animals (Munar et al., 2015), facilitated a conceivable notion of an evolutionary adaptive behavior, possibly developed through the avoidance of the potentially harmful edges (Bar and Neta, 2006). This “threat hypothesis” was backed up by neuroimaging data showing the activation of cerebral areas involved in processing of threat and fear (i.e., amygdala) when viewing greyscale images of edgy everyday objects, as opposed to their curved counterparts (Bar and Neta, 2007). Besides the evolutionary-based approach, other research found that the preference for curvature can also be modulated by trends or Zeitgeist effects (Carbon, 2010). Zeitgeist effects -translated literally as “spirit of the times”–designate time-related fluctuations in values (for instance, aesthetic ones) influenced by societal phenomena. This perspective noted the omnipresence of curvature in current contemporary times, enabled by technological advancements that allow the production of curves in time and cost-efficient ways, highlighting a confounding factor of time-specific preferences. Conversely, a different approach considered that the preference might stem from the shape of the curvature by itself, which provides good stimulus continuity (Wagemans et al., 2012), and thereby answers to one of the main Gestalt principles (Bertamini et al., 2016). A review developed a unifying framework for research on the psychological and neural mechanisms of curvature preference, distinguishing between sensorimotor-based explanations and those originating from appraisals (Gómez-Puerto et al., 2016). The review proposed that the learnt versus evolved/innate origins of the preference are not mutually exclusive, however, they require further research to uncover cultural and evolutionary foundations.

### Contours in interior environments

Extending on the empirical evidence for this suggested contour effect, an encouraging body of experimental literature proposed similar patterns in the context of architecture and interior design. A positive response to curved/curvilinear as opposed to angular/rectilinear spaces was observed when reacting to images representing matched sketches/line drawings (Madani Nejad, 2007), colored (van Oel and van den Berkhof, 2013) or greyscale (Dazkir and Read, 2012) computer-generated scenes, and images of real environments (Vartanian et al., 2013, 2019), in addition to drawings of building facades (Ruta et al., 2019). Studies have shown that curvature was preferred over angularity and resulted in higher self-reported positive emotions such as pleasure (Küller, 1980; Hesselgren, 1987; Dazkir and Read, 2012; van Oel and van den Berkhof, 2013), relaxation, safety, privacy (Madani Nejad, 2007), in addition to a self-reported decision to approach (Dazkir and Read, 2012).

Most of previous studies used subjective semantic scales to depict affective and behavioral responses (e.g., valence, arousal, and approach-avoidance), with recent approaches including neuroscientific measures such as neuroimaging (Vartanian et al., 2013; Banaei et al., 2017). Although earlier research has attempted to cover a wider range of emotional responses, more recent studies have been focused on aesthetic preference measures, such as liking, pleasantness, attractiveness, and beauty. We note however that the main portion of the evidence on the contour effect originates from empirical aesthetics, a discipline highly concerned with the question of hedonic tones. This has been noted as a general limitation of the emerging lines of research investigating the effects of architectural spaces, which are mostly restricted to aesthetics and disregard other components of the cognitive-emotional dimension of architecture (Higuera-Trujillo et al., 2021). Beyond aesthetic preference and hedonic tones, environmental psychologists explore affective responses from additional domains, and highlight a particular role of the environment in regulating emotions and affecting mood (e.g., stress reduction Ulrich et al., 1991), thereby influencing human psychology and physiology.

In terms of stimulus material, previous research mainly adopted traditional presentation methods and used either matched but unrealistic stimuli with a limited number of images [e.g., *N* = 8 in Madani Nejad (2007); *N* = 4 in Dazkir and Read (2012)], or a higher number of images of real environments at the (substantial) cost of accepting a considerable number of confounding factors (Vartanian et al., 2013, 2019), adding in both cases further limitations to the generalizability of results. Research investigating objects, on the other hand, ensured matched stimuli, and presented greyscale photographs of real objects (Bar and Neta, 2007; Cotter et al., 2017) or line drawings (Chuquichambi et al., 2021; Sinico et al., 2021). However, to the best of our knowledge, all previous studies were typically restricted to one image per environment/object, thereby showing stimuli exclusively from one side. It is worth noting that the subject has received little experimental scrutiny beyond traditional stimulus presentation methods (i.e., static images), with very limited endeavors adopting real life objects/environments or virtual reality (VR) to reflect the three-dimensional experience. When comparing with traditional presentation modes, evidence on the curvature effect in virtual environments seems inconsistent. Empty virtual rooms with curved boundaries were found to elicit more pleasantness and arousal than those with linear boundaries (Banaei et al., 2017), while no effects were observed in another study where participants were immersed in photo-realistic virtual interiors (Tawil et al., 2021).

Additional (heterogeneous) evidence is emerging with extended research efforts and attempts to uncover the underlying psychological and neuronal mechanisms of this positive effect of curvature in interior contexts. While neuroimaging data resulting from an investigation of everyday objects demonstrated an activation of the amygdala when individuals perceive edgy stimuli (Bar and Neta, 2007), this was not observed with interior design stimuli (Vartanian et al., 2013), Conversely, curvilinear environments activated the medial orbitofrontal cortex. Subsets of the same image set were used in following studies yielding inconsistent effects, with the latest one finding a preference for rectilinear over curvilinear interiors (Palumbo et al., 2020). Interestingly, in the same study, curved abstract shapes were still preferred over angular ones. Of note, unlike the majority of previous research, in this study participants were mostly men.

Indeed, recent evidence suggested that the positive curvature effects might be moderated by individual factors such as gender and academic degree, highlighting that most of the findings from previous studies relied largely on female psychology students (Palumbo et al., 2021). Earlier research, however, has identified sex differences, linking contour preference and sketch production to symbolic representations of the human body morphology (Munroe et al., 1976). Similar tendencies were also observed in a previous study from our lab, where a significant positive effect of angular rooms (on cognitive performance and subjective ratings of affect and spatial experience) was found in male when compared to female participants (Tawil et al., 2021). However, to date, no study has yet attempted to examine these differences in contours evaluation with interior design stimuli.

### The present study

Within the scope of the present study, we aimed to investigate the response to contours in interior environments, while addressing some of the limitations of previous research. Given that our earlier investigation of these effects in VR returned null results, we opted to test our stimulus material under the traditional presentation paradigm (i.e., presenting 2D static images), similarly to the biggest portion of previous studies. However, we provided more than one perspective of the same environment. Eventually, we presented 20 well-matched photo-realistic images representing a contrast in contours (angular vs. curved). We included style (modern vs. classic) as a second-level factor to take into consideration the evidence on a Zeitgeist effect potentially moderating curvature preference (Carbon, 2010). For the purpose of exploring internal processes possibly responsible for the assumed positive effects of curved contours beyond mere preference, we distinguished between aesthetic and stress responses. Aesthetic preference was represented by self-reports on beauty and liking, two measures that were mostly used in previous research. Stress response, on the other hand, was explored through the lens of the basic physiological antagonism parasympathetic – sympathetic activation, therefore, we included subjective evaluations of rest and stress. Moreover, we took the decision (after pre-registering) to control for a balanced sample in terms of reported biological sex in order to identify any potential differences. We expected a positive impact of curved contours on the explicit responses collected *via* subjective ratings of aesthetic preference (i.e., higher beauty and liking scores) and stress response (i.e., lower stress and higher rest scores). Conversely, considering the scarcity of evidence in the literature, we did not have a strong *a priori* prediction regarding any of the interactions of contour with style and/or biological sex.

## Materials and methods

### Participants

Based on unpublished results from a previous study piloting an implicit task using similar stimuli as in the present study (i.e., static images), a sample size estimation using G*Power—version 3.1 (Dusseldorf University, Dusseldorf, Germany) resulted in the need for 138 participants to enable a small effect size (*f* = 0.10) with an alpha of 0.05 and a power of 0.80 for a within-subjects repeated measures ANOVA. Due to the potentiality of technical errors (or abortion mid-experiment), we aimed for a sample of up to 200 participants, to obtain at least 150 full datasets. The additional sample buffer was considered because the *apriori* effect size was based on one experimental task only. Recruitment was carried out *via* the online platform Prolific (www.prolific.co), and was stratified by sex (50:50). Eventually, 198 healthy adults were included in the study (aged between 18 and 69 years, *Mdn* = 27.0 ± 10.9; 50% female participants), with no severe/uncorrected visual impairments. Further in−/exclusion criteria included fluency in German and self-reported absence of diagnosed mental or neurological disorder. Subjects were compensated with 8£ for participating in all parts of the experiment, which lasted for approximately 1 h in total. For further sample characteristics, see Table 1.

**Table 1:** Sample characteristics (*N* = 198).

	Range^a^	*M*	*SD*	Freq.	*%*
**Biol. variables**					
Median age	18–69	27.0	10.9	–	–
Self-reported biological Sex (male/ female)^b^		–	–	99/99	50/50
**Net income**					
<1.250	–	–	–	84	42.4
1.250–1749	–	–	–	30	15.2
1.750–2.249	–	–	–	16	8.1
2.250–2.999	–	–	–	28	14.1
3.000–3.999	–	–	–	13	6.6
4.000–4.999	–	–	–	6	3.0
>5.000	–	–	–	8	4.0
do not want to answer	–	–	–	13	6.6
**Education**					
Median years of education^c^	5–13	12	1.33	–	–
Nominal level of education^d^					
No school degree	–	–	–	1	0.5
Low school degree	–	–	–	2	1.0
Middle school or lower	–	–	–	18	9.1
Highschool (A-levels)	–	–	–	177	89.4
**Architectural/aesthetics knowledge**					
Profession architecture/ interior design – yes	–	–	–	5	2.5
Median VAIAK – interest^e^	11–74	37.0	14.5	–	–
Median interior design interest VAS^f^	0–100	61.0	27.5	–	–
Median interior design – knowledge VAS^f^	0–100	23.0	23.8	–	–
**Psychopathology**					
Median DASS21- stress^g^	0–36	10.0	7.24	24	12.1
Median DASS21 – anxiety^g^	0–28	2.0	5.32	41	20.7
Median DASS21 – depression^g^	0–42	4.0	7.95	50	25.3

a Observed value range.

b The terms “male” and “female” are used as grouping adjectives, as this was how participants were asked to (dichotomously) classify themselves.

c School and professional education.

d Based on German education system.

e VAIAK, Vienna Art Interest and Knowledge Questionnaire, interest subscale, total scores can range from 1 to 77, in the original validation study, the mean for lay people was *M* = 37.9, SD = 12.9 (Specker et al., 2020).

f Visual analogue scale (0–100) to rate interest or knowledge concerning architecture and interior design.

g Values under frequency column are the number of subjects reaching a clinically meaningful cut-off (i.e., moderate severity) on the DASS21, depression, Anxiety and Stress Scale 21. The terms “male” and “female” are used as grouping adjectives, as this was how participants were asked to (dichotomously) classify themselves.

### Stimulus material

**Figure 1:**
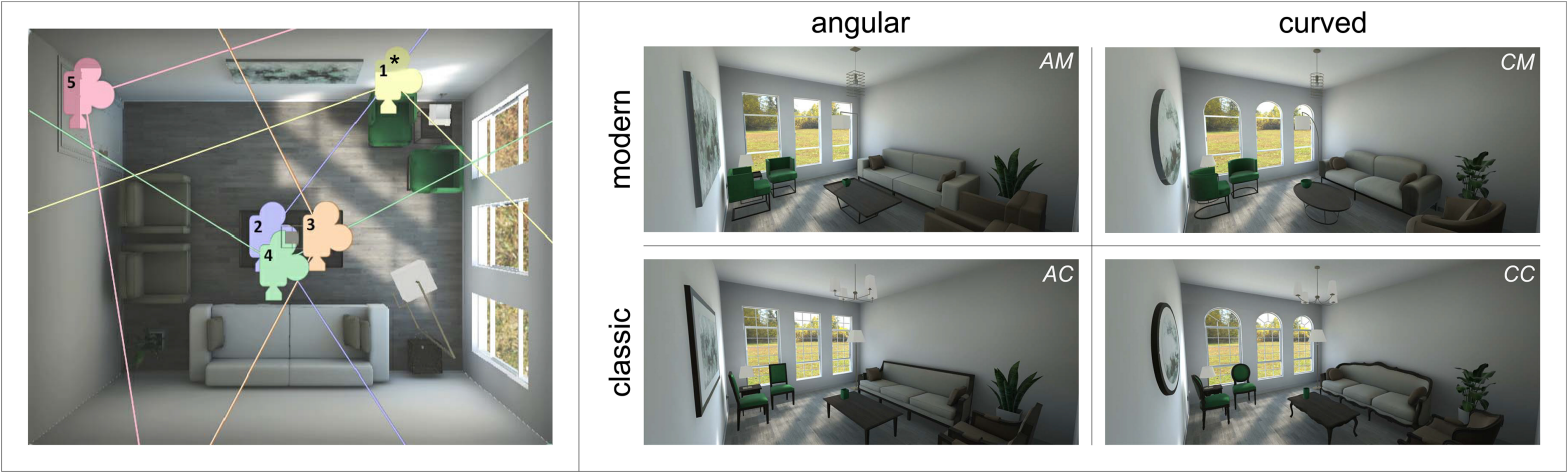
Stimulus material. Left: Plan of the room illustrating the five viewpoints/perspectives, numbered according to the order of presentation (angular modern condition is displayed as a room example). All cameras were positioned to simulate eye view from standing position (approximately 1,500 mm), except for camera 1 (marked with a star (*)) which was placed to replicate a view from a sitting position. Right: Example stimuli representing the 2×2 design and depicting “perspective 5” in all four conditions: angular modern (AM), curved modern (CM), angular classic (AC), and curved classic (CC).

The stimulus material was derived from a previous study where stimuli were presented in VR (Tawil et al., 2021). Minor adjustments were implemented to achieve further control over possible confounding variables. Two pairs of virtual living rooms were created using Autodesk’s 3ds Max (L × W × H = 4.9 × 3.9 × 3 meters) and implemented in the gaming software Unity (version 2019.2.1f1, 64-bit). Rooms of each pair were identical in their design, except that one had angular objects, while the other had curved counterparts (factor 1 contour: angular vs. curved). The second contrast was the interior design style (factor 2 style: modern vs. classic). Each room included 18 objects that were matched in terms of bounding sizes, materials, and colors, and contrasted according to the study design factors (a comparative list of all objects from all rooms along with their images and dimensions is included in section 1.3 of the Supplementary material). The pairs were designed (by an expert in architecture) with the main objective of providing balanced and proportional objects that still reflect the same design spirit/style, without appearing unrealistic or unfamiliar. Therefore, they were inspired by common furniture that exist in both contour versions. In terms of style, we intended a periodic contrast rather than one relating to specific aesthetics, in order to investigate the previously proposed Zeitgeist effect (Carbon, 2010). However, to discriminate between the styles, the “classic” pair had items that originate from more traditional design periods (e.g., “Rococo” Louis XV furniture, “neoclassical” Louis XVI furniture, and “Georgian” sliding slash windows), while the “modern” one included items inspired by “minimalism,” a much more recent style characterized by simplicity and clean lines. To provide diversity in the stimulus set, different cameras were placed inside the virtual rooms to capture different viewpoints from a first-person perspective. Images were rendered using Unity High Definition Render Pipeline (HDRP, version 6.9.1), and captured within Unity using the tool “Screenshot Utility”,^1^ downloaded *via* the Unity Asset Store. Image size was set to 5,075 × 2,160 pixels, 4 K resolution with ratio 21:9. Since we aimed to control for low-level image features [using ImageDecomposer^2^ provided by Berman and colleagues (Berman et al., 2014; Kardan et al., 2015)], eventually, five out of the 15 generated images were selected per room, capturing all angles (for details on the low-level feature values and t-tests to compare curved vs. angular and modern vs. classic stimuli, please refer to section 1.1 of the Supplementary material) and a total of 20 images were included in the final stimulus set (the virtual cameras’ positions are shown in Figure 1 for each of the perspective views). Each image belonged to one of the four categories: angular modern (AM), curved modern (CM), angular classic (AC), and curved classic (CC). Examples of the stimuli used are shown in Figure 1 (refer to section 1.2 of the Supplementary material for the complete stimulus set).

### Experimental design and procedure

**Figure 2:**
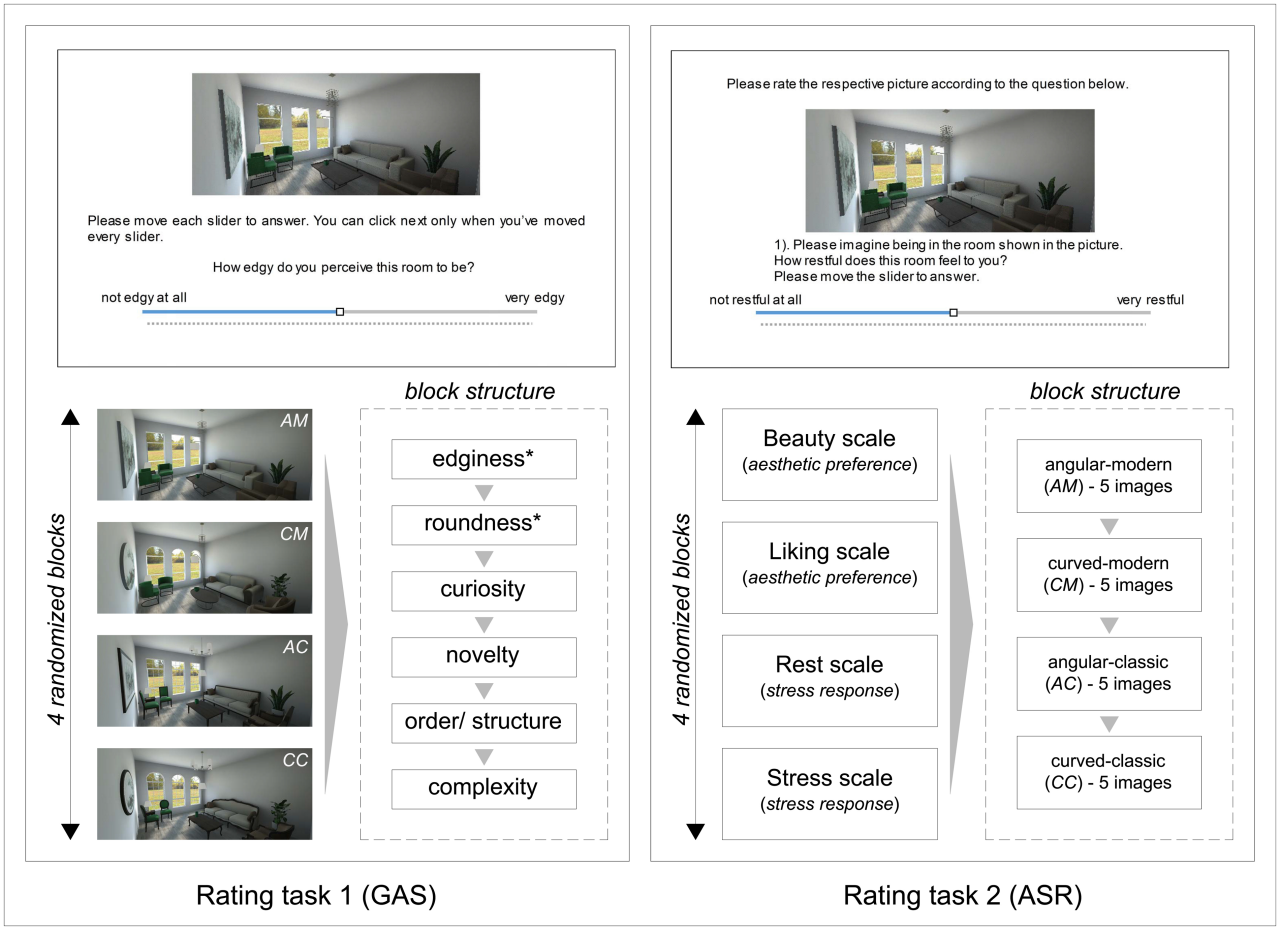
Illustration of the rating tasks structure. Left: Rating task 1 – General appraisal scale (GAS), consisting of four randomized blocks, each including six ratings (DV) of the same image (IV). Dimensions marked with a star (*) were meant for manipulation check and were reported within the present analyses. An example slide is illustrated on top of the structure, depicting “edginess” rating. Right: Rating task 2 – Aesthetic and stress response (ASR), consisting of four randomized blocks, each including ratings for 20 images (IV) on one single dimension (DV). The example slide shows “rest” rating scale. Both example trials are reconstructed and translated from German for illustration purposes.

The experiment was implemented online using Inquisit 6 (millisecond, 2021), and a link to the study was provided for the participants on the online-recruitment-platform Prolific,^3^ with a completion code shown at the end for collection of the monetary compensation. Subjects were first presented with the study information, asked for their informed consent, and answered questions concerning the eligibility criteria. The experiment included three main sections. First, participants responded to a set of four different reaction time paradigms (1) *approach-avoidance task* (AAT; Wiers et al., 2011), (2) *implicit association task* (IAT; Greenwald et al., 1998), (3) *dot-probe* (DP; Bradley et al., 1992), and (4) *manikin task* (MT), (De Houwer et al., 2001)] that were intended to capture implicit responses, but which are not part of the current paper. Second, participants filled out questionnaires (only those reported in demographics later on in this paper are fully cited here) assessing socio-demographic details, general interest/knowledge in arts and architecture (adapted from The Vienna Art Interest and Art Knowledge Questionnaire, VAIAK; Specker et al., 2020), preferred interior design styles, tendencies to depression, anxiety, and stress to check psychopathology levels (DASS-21; Lovibond and Lovibond, 1995), and personality traits, in addition to information about growing up, current housing conditions and exposure to nature. Within the socio-demographic questionnaire, participants were also asked to indicate their biological sex (male or female), thus, for reasons of simplicity, we will be using the term “sex” when referring to related potential differences, and the adjectives “male” and “female” for the subgroups of participants who indicated either category. In the third part, which constitutes the main focus of the present analysis, participants responded to two sets of rating tasks, created for the purpose of this experiment. Each set was composed of four blocks, randomized across participants. Responses were collected on visual analogue scales (VAS, 0–100, numbers were invisible to participants to avoid direct comparisons) anchored with statements on both endpoints and shown below the to-be-rated image (for details on the questions in German language, see Supplementary Table 1). Images were set to 50, 50% (height, width) of their original size and placed at the center of X (50%) and slightly upwards (30%), relative to the screen size of participants (example slides are shown in Figure 2 below).

#### Rating task 1 – General appraisal scale (GAS)

In each of the GAS blocks, participants rated the four images displayed in Figure 1 (AM, CM, AC, CC; depicting a general perspective view from the door) on six dimensions representing a general spatial evaluation (VAS scales 0–100). Thus, a total of 4 (images) × 6 (rating dimensions) = 24 ratings were completed by each participant. The order of the rating dimensions was kept identical across blocks (i.e., edginess, roundness, curiosity, novelty, order/structure, and complexity), but images were presented in random order (refer to Figure 2 below). We will only report ratings on edginess and roundness, as these scales were intended as a stimulus manipulation check. The items (translated from German) were for *edginess* “How edgy do you perceive this room to be?” (left anchor: [0] “not edgy at all,” right anchor: [100] “very edgy”), and for *roundness* “How round do you perceive this room to be?” (left anchor: [0] “not round at all,” right anchor: [100] “very round”).

#### Rating task 2 – Aesthetic and stress response (ASR)

Rating scales of the ASR represented “aesthetic preference”, namely, *beauty* and *liking,* that were mostly used in previous studies on contours [e.g., (Vartanian et al., 2013; Palumbo et al., 2020)], in addition to “stress responses”, operationalized to resemble basic psycho-physiological states in the form of self-reports on *rest* and *stress* (adapted from Madani Nejad, 2007). Blocks of the ASR scale were each related to one different dimension (beauty, liking, stress, rest), and presented in randomized order. Within every randomized block, participants rated each of the 20 stimuli, always presented in the same order, hence a total of 80 responses were collected from each participant (20 trials × 4 blocks) (refer to Figure 2). The items (translated from German) were for *liking* “How much do you like the room shown in this picture” (left anchor [0] “not at all,” right anchor [100] “very much”), for *beauty* “Please rate the beauty of the room shown in this picture” (left anchor [0] “not beautiful at all,” [100] “very beautiful”), *rest* “Please imagine being in the room shown in the picture. How restful does this room feel to you?” (left anchor [0] “not restful at all”; right anchor [100] “very restful”), and for *stress* “Please imagine being in the room shown in the picture. How would you describe your emotional reaction?” (left anchor [0] “relaxed”; right anchor [100] “stressed”).

### Data analysis

We preregistered our research plan (which can be retrieved from https://aspredicted.org/B65_HP6) before the start of the study, as part of a larger experiment that adopted a novel approach using a battery of implicit tasks. However, due to the complexity of the experiment, we find it a crucial initial step to first explore, discuss, and report explicit responses to be able to relate the present study to previous ones with explicit assessments and to interpret any potential effects found through the reaction time paradigms. Although the explicit measures were not detailed in the preregistration, a general preference for curved over angular shapes was assumed, which would also be reflected in explicit rating differences of the stimulus material; i.e. higher aesthetic ratings, and lower stress as well as higher rest ratings for curved vs. angular stimuli.

All data analysis was conducted using R Studio—v1.4 Tiger Daylily (RStudio, Boston, MA, United States).

We split the analysis into three parts, matching the logic of our research questions, and following both a theory- and data-driven stepwise approach. The dependent variables (DVs) were participants’ responses on the 0–100 VAS scales. Mean scores were assessed for each dependent variable (i.e., rating dimension) *via* repeated-measures analysis of variance (RM ANOVA). All analyses were controlled for repetitions within participants by means of the factor “subject.”

We first conducted a manipulation check to test the contrast validity of our stimulus set (level 0). Thereby, to confirm the contour contrast was well discriminated within both styles, participants’ ratings on “edginess” and “roundness” were analyzed separately following two 2 (contour: angular vs. curved) × 2 (style: classic vs. modern) repeated-measures ANOVAs. One dataset was excluded from the analysis due to missing values (*N* = 197 subjects included), and a total of 788 observations (197 participants × 4 images) were included in the analysis of each of the two rating scales (total = 1,576 data points).

For the main analyses, we conducted four two-way repeated measures ANOVA for each of the rating dimensions of the ASR scale to compare the main effects of contours (angular vs. curved) and style (classic vs. modern) as within-subject factors [IVs], as well as their interaction effects on the aesthetic (beauty, liking) and stress (rest, stress) response rating scores [DV] (level 1). Although we were interested in the overall response to the rooms rather than to each individual frame, we did not aggregate scores across perspectives prior to conducting the tests, and total of 3,960 data points were included in each of the four models (198 participants × 20 images). Since the effect of style exclusively was not part of the research questions addressed in this paper, related main effect analyses are briefly described within the manuscript (but are included in detail in Supplementary Table 9).

In the following step, and as we intended to examine potential sex differences (see introduction section for details), we conducted separate mixed ANOVAS for each of the dimensions of the ASR, with contours (angular vs. curved) as a within-subjects factor and sex as a between-subjects factor and as moderator variable [i.e., interaction effects]) (level 2).

For all models, we first report the main omnibus effects then interactions (with the respective descriptive statistics and effect sizes), each followed by the related pairwise comparisons on the different stimulus factors corrected using the False Discovery Rate method (FDR; Benjamini and Hochberg, 1995), along with effect sizes estimated by means of Cohen’s d (Cohen, 1988). According to the commonly used interpretation, effect sizes are referred to as small (*d* = 0.2), medium (*d* = 0.5), or large (*d* = 0.8). We used the package “afex” (Singmann et al., 2022) to fit the models and produce inferential statistics, package “emmeans” (Lenth et al., 2022) for the pairwise comparisons, and package “effectsize” to compute Cohen’s d values (Ben-Shachar et al., 2020).

Furthermore, we performed reliability analyses to check whether the ratings employed served as reliable measurement techniques for the aesthetic and stress responses to contours. For each of the rating scales, the different stimuli were regarded as “items” which were used to calculate Cronbach’s α. As each image was repeated only once within each rating scale, every rating value was considered as one “item.” Using function “cronbach.alpha” from the package “ltm” (Rizopoulos, 2022), Cronbach’s α was calculated separately for each group of stimuli that we expected to produce similar explicit response (separately for each of the four combinations resulting from the 2×2 design).

## Results

### Manipulation check – Level 0

Results of the manipulation check (level 0) confirmed a highly significant main effect of contours on both *edginess* [*F*(1, 196) = 2567.11, *p* < 0.001, η^2^_g_ = 0.83) and *roundness* [*F*(1,196) = 2173.42, *p* < 0.001, η^2^_g_ = 0.82] ratings. Pairwise comparisons showed that images of angular contours were rated as more *edgy* [*t*(196) = 50.67, *p* < 0.001, *d* = 3.62; *M* = 88.96 ± 11.22] and less *round* [*t*(196) = −46.62, *p* < 0.001, *d* = 3.33; *M* = 7.87 ± 10.29] than those of curved ones (*edginess*: *M* = 18.81 ± 14.68; *roundness*: *M* = 77.11 ± 16) with exceptionally large effect sizes.

Significant interactions of contours with style were observed within both *edginess* [*F*(1,196) *=* 10.85, *p* = 0.001, η^2^_g_ = 0.01] and *roundness* [*F*(1,196) = 10.15*, p* = 0.002, η^2^_g_ = 0.01] scales. Post-hoc comparisons revealed that while our sample rated the angular versions of the images equally among the two styles concerning both edginess and roundness, significant differences were observed between ratings of the curved versions when they were depicting a classic as opposed to modern style [*edginess: t*(196) = −4.85, *p* < 0.001, *d* = 0.35; *roundness: t*(196) = 4.19, *p* < 0.001, *d* = 0.30], whereby images of classic style were perceived as edgier and less round than their modern counterparts. However, effect sizes were small (*edginess*: *d* = 0.35; *roundness*: *d* = 0.3), and this did not substantially influence the effects of contour, which remained particularly significant for the two rating dimensions within both styles (*d* > 2.78 for all four comparisons) (see Supplementary Figure 1 for a graphical depiction of main effects of contour and the interaction with style, and Supplementary Tables 2–6 for further descriptives).

### Aesthetic and stress response ratings (level 1)

#### Main effect of contours

The 2 (contour: angular vs. curved) × 2 (style: modern vs. classic) RM ANOVA confirmed a general main effect of contours on all four dimensions of the ASR: *beauty* [*F*(1,197) = 10.09, *p* = 0.002, η^2^*_g_* = 0.01], *liking* [*F*(1,197) = 6.32, *p* = 0.013, η^2^*_g_* = 0.01], *rest* [*F*(1,197) = 99.18, *p* < 0.001, η^2^*_g_* = 0.12], and *stress* [*F*(1,197) = 63.80, *p* < 0.001, η^2^*_g_* = 0.08].

**Figure 3:**
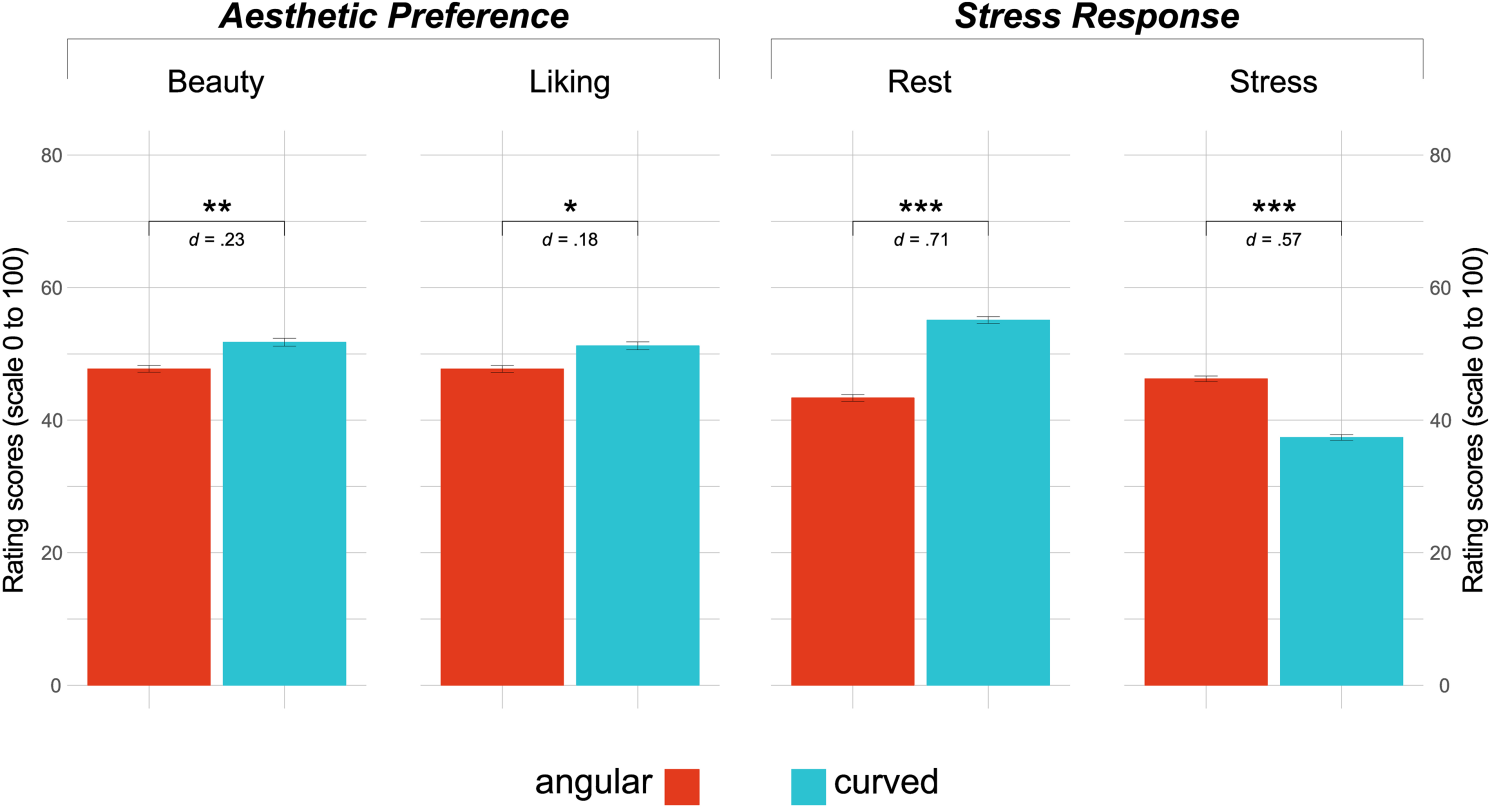
Contour main effect. Left to Right: Results of the analyses comparing mean scores of images of angular vs. curved interiors on the four rating scales representing “aesthetic preference” (*beauty* and *liking)* and “stress response” (*rest* and *stress*) evaluations. Scoring is on a range of 0–100. Bar graphs represent mean scores; error bars indicate standard errors. Asterisks represent significance, *p < 0.05, **p < 0.01, and *** *p* < 0.001.

Pairwise comparisons revealed that images of curved contours were rated significantly higher than those of angular ones on aesthetic preference scales with small effect sizes: *beauty* [*t*(197) = −3.18, *p* = 0.002, *d* = 0.23; curved: *M* = 51.72 ± 14.87, angular: *M* = 47.75 ± 15.07] and *liking* [*t*(197) = −2.51, *p* = 0.01, *d* = 0.18; curved: *M* = 51.22 ± 15.57, angular: 47.68 ± 16.13]. Furthermore, in terms of stress response, images of curved contours scored higher on *rest* [*t*(197) = −9.96, *p* < 0.001, *d* = 0.71; curved: *M* = 55.1 ± 13.30, angular: *M* = 43.34 ± 15.30] and lower on *stress* [*t*(197) = 7.99, *p* < 0.001, *d* = 0.57; curved: *M* = 37.39 ± 11.83, angular: *M* = 46.22 ± 13.62] when compared with images showing angular interiors, with medium effect sizes. Figure 3 depicts the results of the contour main effect (refer Supplementary Tables 7, 8 for further descriptives).

#### Main effect of style

The main effect of style was also significant for all the scales of the ASR (see Supplementary Figure 2 for a graphical depiction of main effects of style, and Supplementary Tables 7, 9 for complete inferential statistics and descriptives).

#### Interaction of contours × style

**Figure 4:**
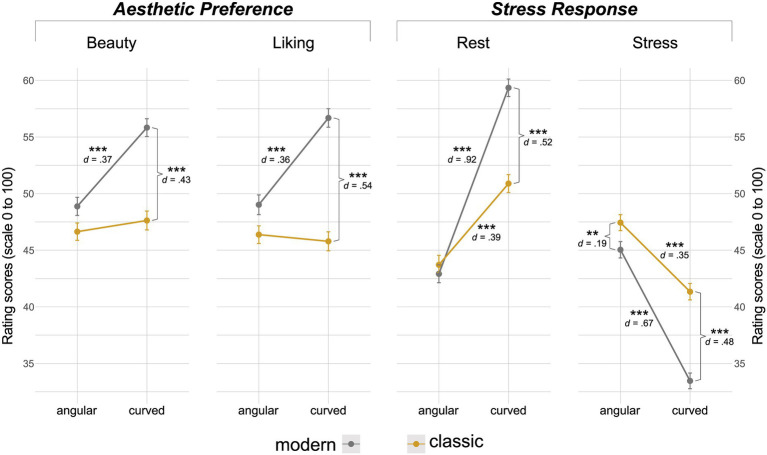
Contours and Style interaction. Left to right: Interaction plots depicting effects of contours, as a function of style, on each of the four rating scales representing “aesthetic preference” (*beauty* and *liking)* and “stress response” (*rest* and *emotion*) evaluations. Scoring is on a range of 0–100. Error bars represent means and standard errors. Lines do not indicate any temporal component between data points. Asterisks represent significance, *p < 0.05, **p < 0.01, and *** *p* < 0.001.

There was a statistically significant interaction of contours with style in all the ASR scales *beauty* [*F*(1,197) = 24.78, *p* < 0.001, η^2^*_g_* = 0.01], *liking* [*F*(1,197) = 45.75, *p* < 0.001, η^2^*_g_* = 0.01], *rest* [*F*(1,197) = 85.25, *p* < 0.001, η^2^*_g_* = 0.02], and *stress* [*F*(1,197) = 24.89, *p* < 0.001, η^2^*_g_* = 0.01]. Effects of contours as a function of style are shown in Figure 4 (refer to Supplementary Tables 7, 10, 11 for further descriptives).

Interestingly, in terms of “aesthetic preference”, post-hoc pairwise comparisons revealed statistically significant differences with small effect size only within the *modern style*, whereby *beauty* [*t*(197) = −5.21, *p* < 0.001, *d* = 0.37] and *liking* [*t*(197) = −5.07, *p* < 0.001, *d* = 0.36] scores were significantly higher for curved conditions (*beauty*: *M* = 55.82 ± 16.06; *liking*: *M* = 56.66 ± 16.87) as opposed to angular ones (*beauty*: *M* = 46.87 ± 17.01, *liking*: *M* = 49.00 ± 19.32). No significant differences were observed between images of angular and curved contours within the *classic style* in any of the two scales (*p* > 0.05). Interestingly, although insignificant, the direction of the effect was reversed in *liking* ratings, with curved conditions scoring lower than angular ones within the classic style category (refer to Supplementary Tables 7, 10, 11 for further descriptives).

Conversely, concerning “stress response”, significant differences were observed in *rest* and *stress* scores within both *modern* (medium to large effects) (*rest*: *t*(197) = −12.95, *p* < 0.001, *d* = 0.92; *stress*: *t*(197) = 9.43, *p* < 0.001, *d* = 0.67) and *classic* (small effects) (*rest: t*(197) = −5.45, *p* < 0.001, *d* = 0.39; *stress*: *t*(197) = 4.90, *p* < 0.001, *d* = 0.35) conditions. Within the *modern style*, images of curved contours were rated as more restful (*M* = 59.33 ± 14.07) and less stressful (*M* = 33.45 ± 12.77) when compared with those of angular ones (*rest*: *M* = 42.90 ± 16.79; *stress*: *M* = 45.03 ± 15.13). The same applied to the *classic style*, however, the magnitude of the effect was less pronounced (refer to Supplementary Tables 10, 11 for further descriptives).

### Sex-related differences (level 2)

#### Aesthetic preference ratings

ANOVA results showed a statistically significant two-way interaction of sex and contours for the *beauty* [*F*(1,196) = 10.27, *p* = 0.002, η^2^*_g_* = 0.02] and *liking* [*F*(1,196) = 8.7, *p* = 0.004, η^2^*_g_* = 0.02] ratings (refer to Supplementary Table 12). Interestingly, post-hoc pairwise t-tests indicated that the positive effect of curved conditions was only significant and therewith mostly driven by participants who indicated their biological sex as female (referred to as female participants hereafter), who had rated images significantly higher on *beauty* [*t*(196) = −4.56, *p* < 0.001, *d* = 0.33] and *liking* [*t*(196) = −3.90, *p* < 0.001, *d* = 0.28] when they were showing curved (*beauty*: *M* = 53.85 ± 13.11; *liking*: *M* = 53.21 ± 14.09) as opposed to angular interiors (*beauty*: *M* = 45.97 ± 14.23; *liking*: *M* = 45.61 ± 14.45). There were no observed significant effects of contours on the preference ratings of participants who indicated male as their biological sex (referred to as male participants hereafter) (*p* > 0.05) (refer to Supplementary Tables 13, 14 for further descriptives).

#### Stress response ratings

**Figure 5:**
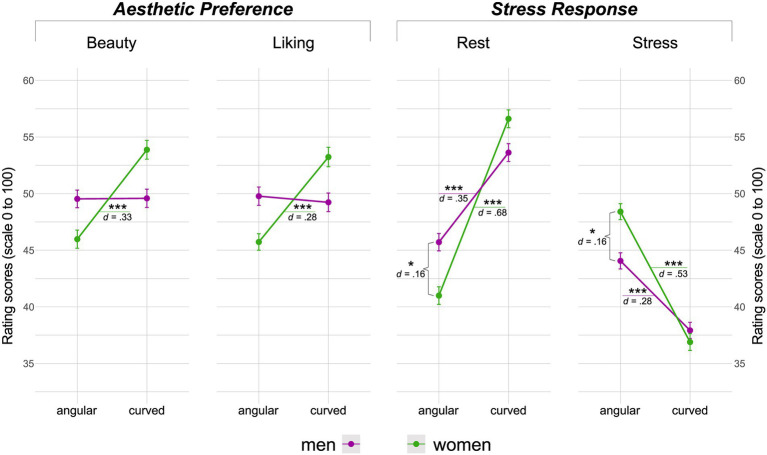
Contours and sex interaction. Left to right: Interaction plots depicting effects of contours, as a function of sex, on each of the four rating scales representing “aesthetic preference” (*beauty* and *liking)* and “stress response” (*rest* and *emotion)* evaluations. The terms “male” and “female” are used as grouping adjectives, as this was how participants were asked to (dichotomously) classify themselves. Scoring is on a range of 0–100. Error bars represent means and standard errors. Lines do not indicate any temporal component between data points. Asterisks represent significance, *p < 0.05, **p < 0.01, and *** *p* < 0.001.

Similar to preference measures, significant interactions of sex and contours were observed in both stress response ratings, namely *rest* [*F*(1,196) = 11.2, *p* = 0.001, η^2^*_g_* = 0.02] and *stress* [*F*(1,196) = 6.06, *p* = 0.015, η^2^*_g_* = 0.01]. However, in contrast with aesthetic preference, the positive effect of curved conditions was found to be significant in both sex groups, although with descriptively lower magnitude in male participants (refer to Supplementary Tables 13, 14 for complete inferential statistics and descriptives). Effects of contours as a function of sex (for both aesthetic preference and stress response) are shown in Figure 5.

### Reliability analysis

The reliability analysis indicated acceptable to good internal consistencies among the different rating scales across all 20 respective stimuli within each of the four stimulus categories (i.e., Cronbach’s α range for *beauty:* 0.80 < α < 0.85, *liking:* 0.8 < α < 0.87, *rest:* 0.74 < α < 0.81, and *stress:* 0.71 < α < 0.81). The results of the reliability calculations are presented in Table 2, along with the respective confidence intervals.

**Table 2:** Reliability of the rating scales (*N* = 198), 5 items (5 picture per category AM, AC, CM, and CC).

	Beauty		Liking		Rest		Stress	
	Cronbach’s α	CI	Cronbach’s α	CI	Cronbach’s α	CI	Cronbach’s α	CI
Angular-modern (AM)	0.82	0.76–0.86	0.85	0.80–0.88	0.80	0.73–0.84	0.75	0.68–0.81
Angular-classic (AC)	0.83	0.78–0.86	0.84	0.79–0.88	0.80	0.74–0.84	0.78	0.72–0.83
Curved-modern (CM)	0.80	0.73–0.85	0.80	0.74–0.80	0.74	0.66–0.80	0.71	0.64–0.77
Curved classic (CC)	0.85	0.80–0.88	0.87	0.83–0.90	0.81	0.75–0.86	0.81	0.74–0.85

Bootstrap 95% confidence intervals (CI) based on 1,000 samples (2.5–97.5%).

## Discussion

In the previous literature, a positive effect of curved as opposed to angular stimuli has been empirically demonstrated, mostly in studies testing images of abstract shapes and (greyscale) everyday objects in different experimental paradigms. However, no consensus has yet been reached as to the source of this preference, with some scholars attributing the preference to attractive intrinsic properties of curves (Palumbo and Bertamini, 2016), while others proposed that it is caused by a possible sense of “threat” elicited by angularity/edginess (Bar and Neta, 2006). A growing body of experimental literature has suggested similar effects in the context of interior spaces and architecture (Dazkir and Read, 2012; Vartanian et al., 2013; van Oel and van den Berkhof, 2013). To investigate this phenomenon in different psychological domains and further examine whether other stimulus- or person-related characteristics can interact with the effect (i.e., interior design style and reported biological sex), we measured the explicit responses to matching photo-realistic images exclusively contrasted in terms of contours (angular vs. curved) and interior design style (modern vs. classic), in a balanced sample in terms of sex (*N* = 198). Building on the evidence in the literature, we hypothesized a positive impact of curved contours on both explicit aesthetic and stress responses collected *via* ratings of beauty, liking, rest, and stress (higher beauty, liking, and rest, and lower stress).

### The effects of contours on aesthetic preference ratings

In line with our hypothesis, our results revealed that contour was a significant predictor for the variability in aesthetic response ratings. The post-hoc results showed that participants, in general, preferred images of curved contours, as indicated by the higher ratings on the liking scale, and found them more beautiful than those showing angular ones. These results support previous findings (Bar and Neta, 2006; Dazkir and Read, 2012; Vartanian et al., 2013, 2019; van Oel and van den Berkhof, 2013) and provide additional evidence on the effects of contours on aesthetic evaluations.

Despite the statistically significant main effects of contours in explaining the variability of aesthetic preference ratings, the percentage of explained variance was considerably low (i.e., 1% for *beauty* and *liking*) suggesting that factors other than contours may play a stronger role in the aesthetic response. In fact, both objective (characteristics of stimuli) and subjective (characteristics of context) factors are proposed to be important in shaping aesthetic experiences (Chamberlain, 2022). In a recent meta-analysis, the first to inspect the consistency of the curvature preference hypothesis, factors other than perceptual contour properties were identified as moderators of the effect, namely, presentation time, stimulus type, expertise, and task (Chuquichambi et al., 2022, pre-print). The study found small to non-significant effects with spatial design stimuli as opposed to larger effects with meaningless or real object stimuli. It might be that the sensitivity to curves in architectural settings involves more complex processes influenced by familiarity, meaning/ affordances, or other observer-related differences. Although we collected information on expertise, only 2.5% of our sample were identified as experts (had a training/profession in architecture/interior design), thereby not qualifying to run any moderation analyses.

Moreover, our results revealed a significant interaction effect between style and contours, confirming the idea of contextual factors other than contour shape *per se* influencing the evaluation. This is consistent with a previous study in which two pairs differing in their styles yielded significantly different self-reported pleasure and approach responses (Dazkir and Read, 2012). Interestingly, when looking at contours within each of the styles separately, results showed that the positive evaluation of curved versions on beauty and liking scales was conditional to the interiors belonging to the modern style, with no significant differences observed within the classic style category. This suggests that, although contours played a general role in aesthetic preference, the effect was dependent on other contextual factors, i.e., in this case style, which explained marginally larger proportions of variance (1 to 2%). Indeed, our sample preferred images of modern over classic style, and rated them significantly higher on the beauty and liking scales. The generally less favorable ratings of classic style might have affected scores and masked the contour-effects. At first glance, these results could be interpreted as being in accordance with previous findings which suggested that in addition to the proposed biological inclination towards curved objects, this preference could also be partly modulated by fashion, trends or Zeitgeist effects (Carbon, 2010). A confounding factor of time-specific preferences was suggested, since recent studies demonstrating a favoring of curved designs have been conducted in a period where curvature has been frequently used. Although we have not instructed our participants to evaluate the images as if they perceived them from a historical perspective, a similar Zeitgeist effect is to be expected when considering the style contrast of our stimulus set. However, the findings cannot confirm whether the observed effects strictly relate to time-specific aesthetics, or are rather the result of the generally negative appreciation of the classic category, or both, a question which would require investigating additional variations.

### The effects of contours on stress response ratings

Generally, contour was a significant predictor of the variability in stress response ratings. Effects were robust and consistent. Pairwise comparisons showed that participants rated images of curved contours as more restful and less stressful than their angular counterparts. In line with previous findings (Madani Nejad, 2007; Dazkir and Read, 2012), our results provide evidence for the relaxing effect of curved contours in interior environments.

Contrary to the findings for aesthetic preference, the curvature positive effect was not dependent upon style, and the factor contour explained larger proportions of variance (8% for *rest* and 12% for *stress* as opposed to 1–2% in the case of aesthetic response). Although there was a significant interaction effect of contours with style, when examining the explicit stress response in rest and stress scales, curvature had a significant, similar positive effect on ratings in both interior design styles (i.e., higher rest and lower stress). The style comparisons showed that images depicting classic style were generally more negatively rated on both scales when compared to those belonging to the modern category, however, the contour effect remained significant. This suggests an overall stronger and more consistent effect of contours on stress response as compared to aesthetics, since stress-related findings “survived” the generally less favorable ratings of the classic style. With reference to the biophilia hypothesis and the deriving frameworks suggesting curvature as a biophilic asset in architecture and design (Kellert and Calabrese, 2015; Salingaros, 2015), it has been argued that nature commonly includes more curves than angles, therefore individuals are expected to be naturally drawn to curves (Salingaros, 2015; Coburn et al., 2019). Beyond exclusive preference, researchers have highlighted a role of curvature (and biophilic design *per se*) in reducing physical (bodily) and psychological stress (Salingaros, 2015, 2019). Our results present first evidence for the relaxing effects of curvature within fully controlled, yet ecologically valid settings.

### Sex-related differences

#### Aesthetic preference ratings

When looking into scores of both sex groups separately, we found a significant effect of the factor “sex” for explaining the variability of aesthetic preferences. Specifically, female participants generally liked images of curved contours more than those of angular ones and rated them higher on beauty, while no differences were observed in male participants’ ratings on both scales. This finding is in line with re-emerging implicit evidence suggesting potential differences in the appreciation of contours observed with abstract stimuli (Palumbo et al., 2021) and virtual indoor environments (Tawil et al., 2021). In fact, earlier research had examined sex differences in preference and production of shapes, and had associated those with “sex-linked symbolic properties” of the stimuli (Munroe et al., 1976). The last study to report sex differences investigated wrapped candies in children (Munroe et al., 1976). Although generally children from both sex groups (*N* = 175) chose the spherical candy over the cube shaped one more frequently, girls chose it even significantly more than boys (83% vs. 57%). The authors linked the effect to one’s conception of their own body, at least regarding objects to be ingested. However, considering the age range of the study population (4 to 12 years old), it could be argued that the results rather speak for “projected body ideals,” as body curves of both male and female sexes are thought to be similar until teenage years. Associations between curvature and femininity were previously proposed in spatial design, whereas sketches of interior spaces were found to be rated higher on the “masculine-feminine” scale as levels of curvature increased (Madani Nejad, 2007). More recent works noted that the main portion of the evidence on the effects of contour in most domains generally stems from homogeneous samples (i.e., female psychology students) (Palumbo et al., 2020), and indeed, a subsequent study observed a stronger preference for meaningless curves within this specific population (Palumbo et al., 2021). The findings were interpreted as evidence that the preference for curves has both social and biological roots. Generally, it has been suggested that men and women vary in how they respond to aesthetics (Djamasbi et al., 2007). Such differences could be related to social norms and gender stereotypes, but also to more biological sex differences (Lueptow et al., 1995). Biologically, sex-related differences in the neural correlate of beauty have been previously demonstrated, with the observed different strategies used for assessing aesthetics attributed to a division of labor between male and female hunter-gatherer hominin ancestors (Cela-Conde et al., 2009). Although our results present the first confirmatory evidence on sex-related differences in the aesthetic preference of curved interiors, the findings do not allow to discriminate whether these effects are sex- or gender-related. Since our sample reported on biological sex, we are using the term “sex,” however, further research is needed to clarify whether these effects result from social constructs related to gender, or are rather intrinsic.

#### Stress response ratings

Conversely, in terms of stress response, our results showed that the factor “sex” did not have a substantial effect on the significance of any of the ratings scores on rest and stress dimensions. Both sex groups rated images of curved contours higher on rest and lower on stress when compared with those showing angular ones. However, the magnitude of the effect was descriptively higher in the female as opposed to male subgroup. Overall however, we observed more consistent effects than in aesthetic preference ratings, with larger effect sizes. The results imply that contours could have a more global effect on the explicit stress response. When comparing the ratings of the two subgroups within each contour category, descriptively, female participants rated images of angular contours more negatively, and those of curved contours more positively when compared with male participants. However, the differences only reached significance in the case of angularity, specifically in both stress response ratings.

In sum, whereas curvature was found to be aesthetically preferred over angularity, this explicit preference was conditioned by the factors “style” and “sex.” In contrast, curvature’s positive effects on explicit stress responses were not dependent on other stimulus- (i.e., style) or individual (i.e., sex-related) factors. The amount of explained variance by contour was considerably higher for stress as opposed to only small amounts explained for aesthetics. Moreover, post-hoc results showed small effect sizes in aesthetic preference ratings compared to those found in the stress response evaluations (medium to large effects). We interpret the independence of the curvature positive effect on the stress responses from context (style) and reported biological sex as hinting towards a generalized, hence perhaps adaptive, phenomenon. Future efforts could examine more implicit mechanisms that present objective indicators of a stress-reduction effect.

Before concluding our discussion, it is worth mentioning that although we observed an effect of curvature when presenting two-dimensional static images of the rooms, the same environments experienced in 3D and in real human scale *via* immersion in VR did not elicit any differences on a large set of affective and cognitive measures, including similar ratings as in the present study. VR has been proposed as an alternative to the costly real life setups, as it allows the manipulation and control of relevant experimental parameters (Franz et al., 2005), while providing the opportunity to enable a feeling of presence in a space, evoking responses that are similar to those elicited by real environments (Bishop and Rohrmann, 2003; Villa and Labayrade, 2012). This is particularly important within the increasing acknowledgement of the role of the body in the architectural experience (Spence, 2020). The present findings reiterate previous concerns as to whether environments affect us in the same way, or ultimately differently, when being inside them as opposed to looking at their image (Nasar, 1994). Although we are not able to directly compare the present results with the ones from our previous study, it is necessary to further explore and compare the curvature effect on the psychological and physiological responses within different presentation modes.

### Limitations and directions for future research

Overall, this paper is far from providing decisive directions, as our current results are limited to explicit responses collected through self-reports, thereby lacking the objectivity required to draw affirmative conclusions. Although our measures lacked a common operationalization of such assessments, we were able to draw initial differentiation on the effects of contour on two different psychological domains that hypothetically operate through different mechanisms. Here, it is worth noting that Cronbach’s coefficients confirmed the reliability of our measures, as they revealed good levels of internal consistencies, especially when considering the low number of “items” (5 items by category). However, given the high cross-correlations between all four rating dimensions (refer to Supplementary Table 15), further research is needed to define the most relevant factors when it comes to rating subjective responses to interior design stimuli, particularly in terms of psychometric scale development. Concerning the results, albeit effects were statistically significant, a small variance was found to be explained by our manipulated factors (contours, style). This implies that other factors may play a stronger role in the aesthetic and affective response variability. Given the complexity of environmental influences, with the present study only tapping into a few aspects of the visual domain, this is somewhat unsurprising. In addition, considering the acknowledged role of affect and/ or inter-individual differences in influencing the response to physical environments, future studies could balance their designs to account for variables such as mood, psychopathology, personality traits, and expertise, among others. As we asked our participants to report on their biological sex and did not assess sociological gender, we are unable to interpret whether the observed effects were the result of sex- or gender-related differences. However, we regard this issue critically and highlight the need to explore such effects beyond the limited perspective provided by the traditional binary definitions. Future works should assess gender identity together with biological sex (e.g., since birth) in a more differentiated way. Although our sample was considerably large when compared to similar studies, the fact that our participants were recruited online may affect the sample representation of a more general population, i.e., the sample was highly educated. Last but not least, with the absence of strong theoretical explanations – which presents one of the main challenges of the emerging fields investigating the psychological impact of architecture and design (Higuera-Trujillo et al., 2021) – it remains necessary to further explore these tendencies within different presentation modes, and with more objective paradigms that can better detect potential adaptive and unconscious responses and indicate more robust evidence on any source of this phenomenon.

### Conclusions

In sum, the present study found differential evidence concerning aesthetic preference and stress response to contours in interior environments. On the one hand, the positive appreciation (beauty, liking) of curved compared to angular contours was found to be context (style) and sex-dependent (i.e., only in modern style, and only in participants who indicated female being their biological sex), suggesting that explicit aesthetic evaluations may vary meaningfully as a function of inter-individual and contextual (perhaps Zeitgeist) effects. On the other hand, the negative effects of angularity and edges on the stress responses (lower rest and higher stress), operationalized to resemble basic physiological/ affective states that may be triggered by environmental contexts, were robust, larger in magnitude, and not style or sex-dependent, also proposing a potentially adaptive response to curves, previously characterized as “biophilic.” To the best of our knowledge, this is the first study that provides such evidence within fully-controlled yet ecologically valid settings (i.e., multiple photo-realistic images representing several perspectives of one space). Taken together, it could be speculated that the effects of contour in interior environments might be more generalizable with respect to psychological and physiological/bodily responses than concerning the more conscious evaluations of aesthetics informed by experience and other cognitive mechanisms. Future works may want to focus on these dimensions which could be more relevant and especially important to informing designs intended for mental health promotion, however, using more implicit measures. On a last note, the significant results observed when presenting the same environments in the form of static stimuli (i.e., images, as opposed to VR immersion) raise the question of which exact role the modes of presentation and immersion play in aesthetic evaluations, stress, and other responses to contours and interior environments *per se* – a question which should be further followed upon.

## Data availability statement

The datasets presented in this study can be found in online repositories. The names of the repository/repositories and accession number(s) can be found at: https://osf.io/mfpk2/.

## Ethics statement

The studies involving human participants were reviewed and approved by Local Psychological Ethics Committee of the psychosocial center at Medical Center Hamburg-Eppendorf (LPEK-0215). The patients/participants provided their written informed consent to participate in this study.

## Author contributions

NT and LA developed the experimental design idea and setup under supervision of SK. NT designed and provided the design stimuli, pre-processed the data, performed the analysis under supervision of LA and SK, and wrote the original first draft of the manuscript. LA and SK edited the first and all subsequent drafts. All authors contributed to the article and approved the submitted version.

## Conflict of interest

The authors declare that the research was conducted in the absence of any commercial or financial relationships that could be construed as a potential conflict of interest.

## Publisher’s note

All claims expressed in this article are solely those of the authors and do not necessarily represent those of their affiliated organizations, or those of the publisher, the editors and the reviewers. Any product that may be evaluated in this article, or claim that may be made by its manufacturer, is not guaranteed or endorsed by the publisher.

## Footnotes

^1^ https://assetstore.unity.com/packages/tools/utilities/screenshot-utility-177723

^2^ https://www.dropbox.com/sh/wykarfxm4jnhda0/AAAx8p3tIFBEqTdLSNIInGOla?dl=0

^3^ www.prolific.co
