# Supplementary material for: The contour effect: Differences in the aesthetic preference and stress response to photo-realistic living environments
Source: Front Psychol. 2022 Dec 1;13:933344. doi: 10.3389/fpsyg.2022.933344 (PMC9752057; doi:10.3389/fpsyg.2022.933344)
Supplement: Supplementary file 1 [file Data_Sheet_1.pdf]

Supplementary Material

1 Supplementary Information

1.1 Stimulus selection based on low-level features

Five low-level feature parameters (mean: edge density, hue, saturation, brightness, entropy) were extracted for each picture in Matlab R2017b using a script (ImageDecomposer, 2014) provided by Marc Berman and colleagues (available at: https://voices.uchicago.edu/bermanlab/stimuli-software/) (for background see Berman et al., 2014 and Kardan et al., 2015). Several rounds of stimulus generation and comparison (between angular vs. curved category pictures using t-tests) were conducted, as our goal was to create two sets of stimuli, that on average would not differ from one another in terms of the above-mentioned image features. Thereby, a simple matching procedure was used. The original picture pool included images taken from 15 different angles from each room (angular – modern, angular – classic; curved – modern, curved – classic), with a total of 60 stimuli. We selected five pictures to capture the rooms fully (i.e., from diverse perspectives), and checked whether there were any significant differences between the dimensions edgy vs. round. The results can be found in Table XX, which were all non-significant for all low-level feature parameters. Effect sizes for the contrast angular vs. curved were all small in magnitude. However, differences between design categories modern vs. classic, albeit all non-significant, were for hue of moderate effect size.

	**T-value**	**p-value (two-tailed)**	**Cohen's d**	**Mean (SD)**	**Mean (SD)**
	Angular vs. Curved			**angular**	**curved**
Hue (mean)	0.583	.567	.261	2.332 (0.315)	2.250 (0.313)
Brightness (mean)	0.136	.893	.061	0.512 (0.079)	0.507 (0.77)
Saturation (mean)	0.216	.832	.096	0.954 (0.027)	0.093 (0.028)
Entropy	0.048	.962	.021	7.392 (0.302)	7.386 (0.310)
Edge density	0.185	.406	.380	0.056 (0.015)	0.051 (0.010)
Green pixels (%)	0.151	.882	.068	0.021 (0.022)	0.020 (0.020)
	Modern vs. Classic			**modern**	**classic**
Hue (mean)	1.220	.238	.546	2.208 (0.307)	2.374 (0.302)
Brightness (mean)	0.967	.346	.433	0.526 (0.076)	0.493 (0.076)
Saturation (mean)	0.896	.382	.401	0.100 (0.030)	0.089 (0.248)
Entropy	0.106	.916	.048	7.396 (0.304)	7.382 (0.308)
Edge density	0.963	.352	.430	0.056 (0.016)	0.050 (0.008)
Green pixels (%)	0.877	.392	.392	0.025 (0.023)	0.017 (0.018)

1.2 Stimulus material

The stimulus material is available at https://osf.io/mfpk2/.

	Angular modern (AM)	***Curved modern (CM)***	***Angular classic (AC)***	***Curved classic (CC)***
1	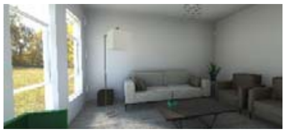	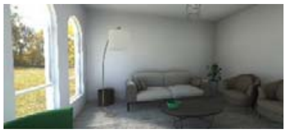	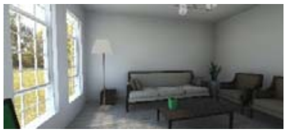	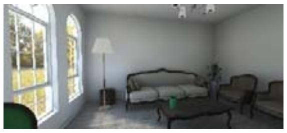
2	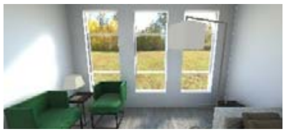	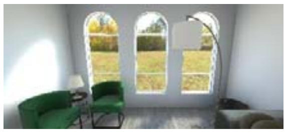	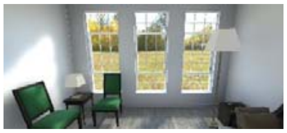	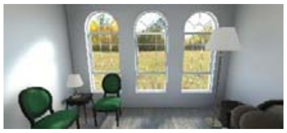
3	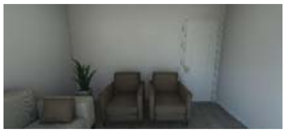	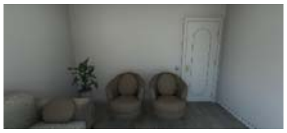	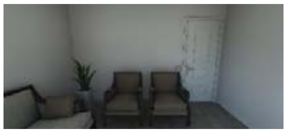	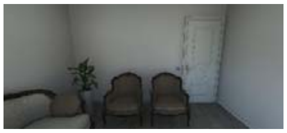
4	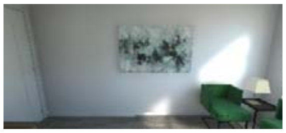	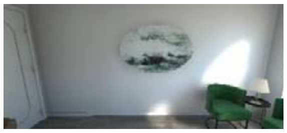	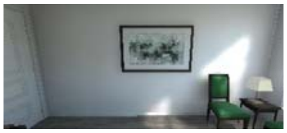	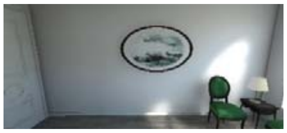
5	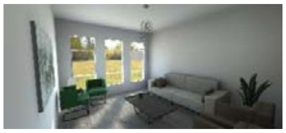	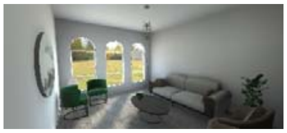	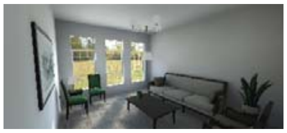	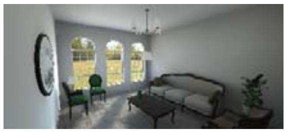

1.3 Inventory of objects and their properties in the different conditions (upper rows depict the modern category, and lower ones show the classic category)

**Furniture**	**(width, height, depth) in meters**	**Angular**	**Curved**
Armchair	(0.8, 0.9, 0.8)	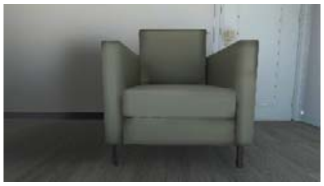	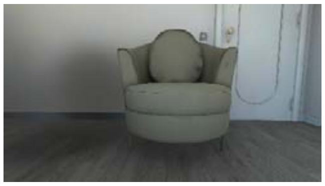
		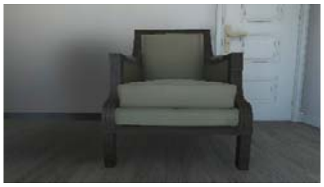	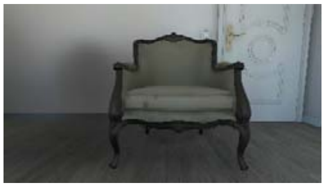

Basket	(0.4, 0.4, 0.4)	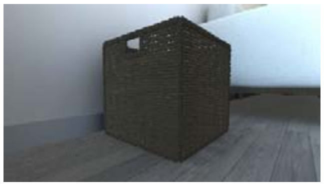	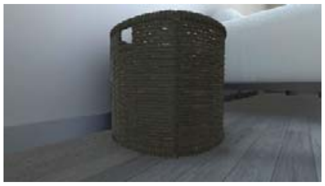
		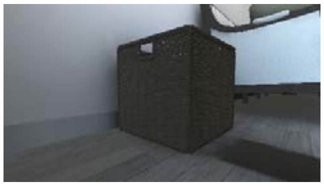	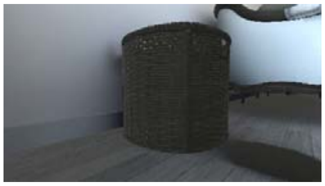
Chair	(0.6, 0.8, 0.6)	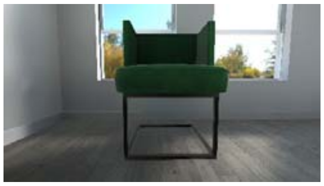	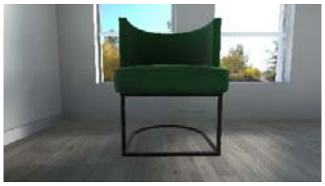
		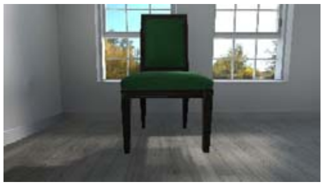	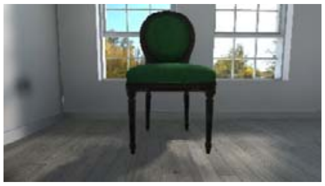
Couch	(2.6, 0.9, 0.9)	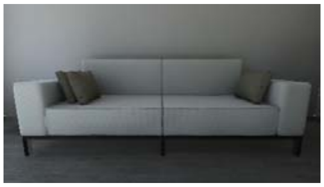	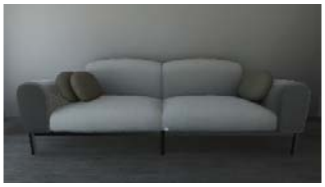
		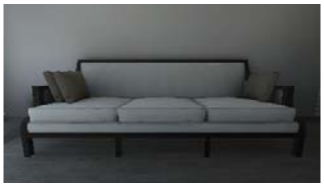	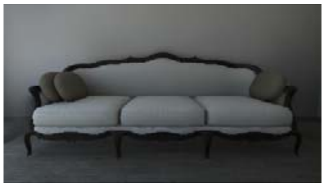
Door	(1.0, 2.3, 0.1)	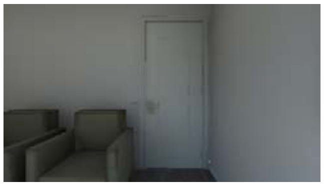	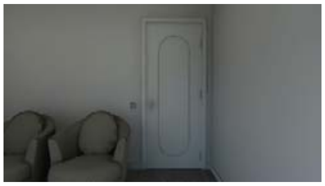
		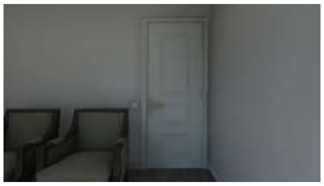	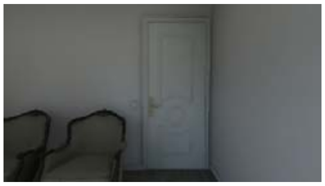

Ceiling lamp	(0.2, 0.8, 0.2)	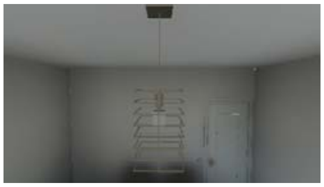	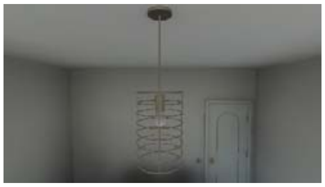
		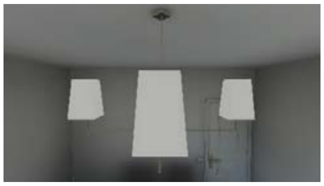	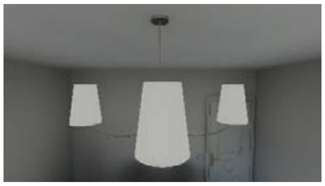
Floor lamp	(1.1, 2.0, 0.4)	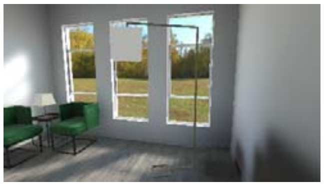	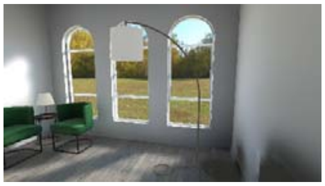
		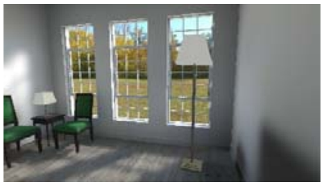	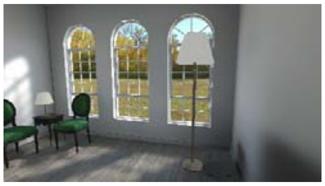
Table lamp	(0.3, 0.4, 0.3)	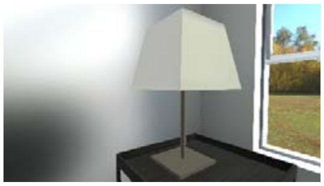	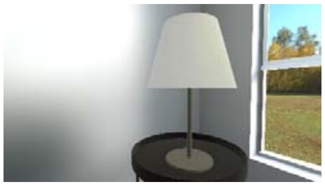
		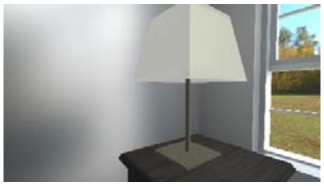	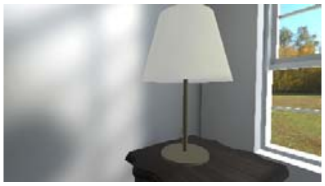
Painting	(1.5, 1.0, 0.04)	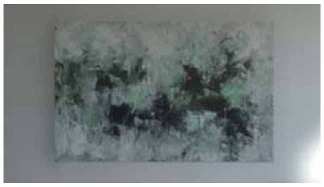	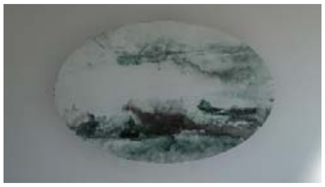

		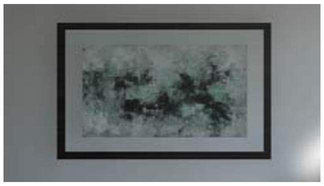	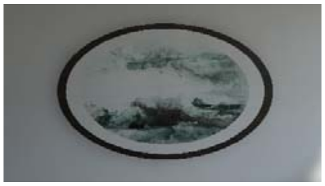
Plant	(0.6, 0.7, 0.5)	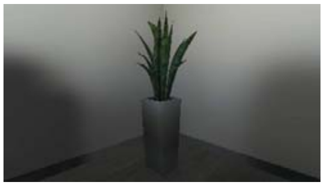	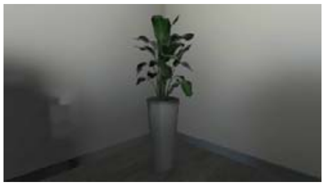
		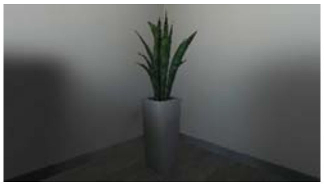	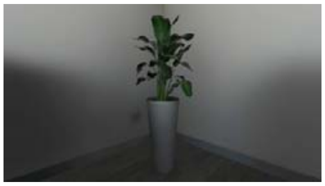
Center table	(1.4, 0.4, 0.8)	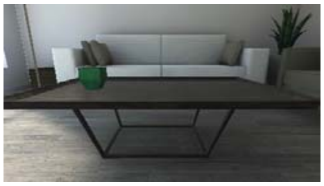	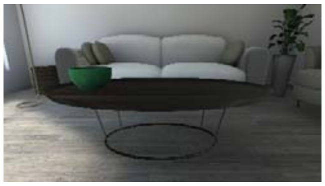
		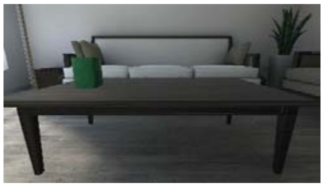	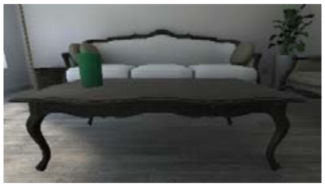
Side table	(0.5, 0.5, 0.4)	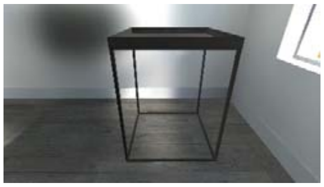	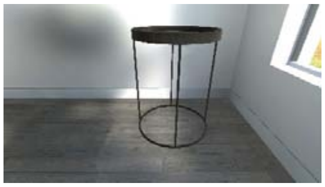
		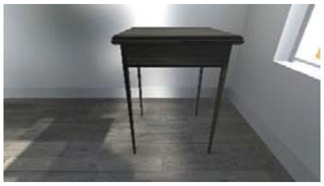	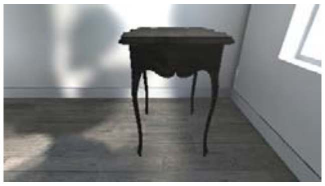
Window	(0.8, 2.0, 0.08)	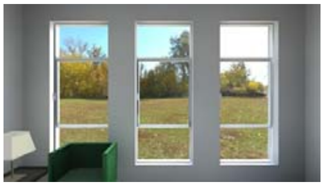	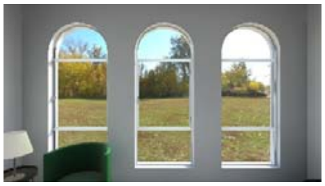

		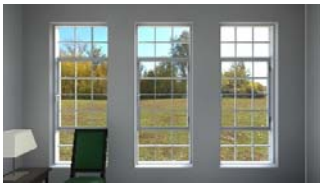	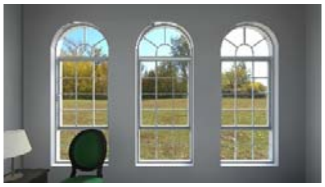
Vase	(0.1, 0.2, 0.1)	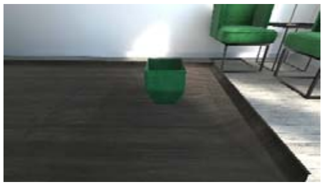	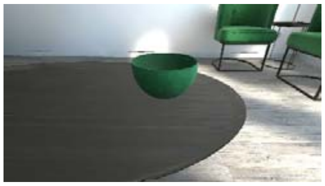
		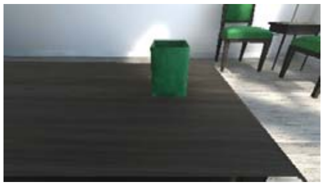	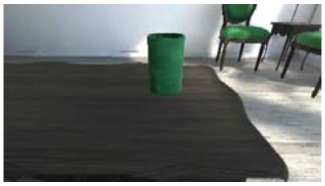

1.4 Data

The data supporting the conclusions of this article is available at https://osf.io/mfpk2/.

2 Supplementary Figures and Tables

2.1 Supplementary Figures

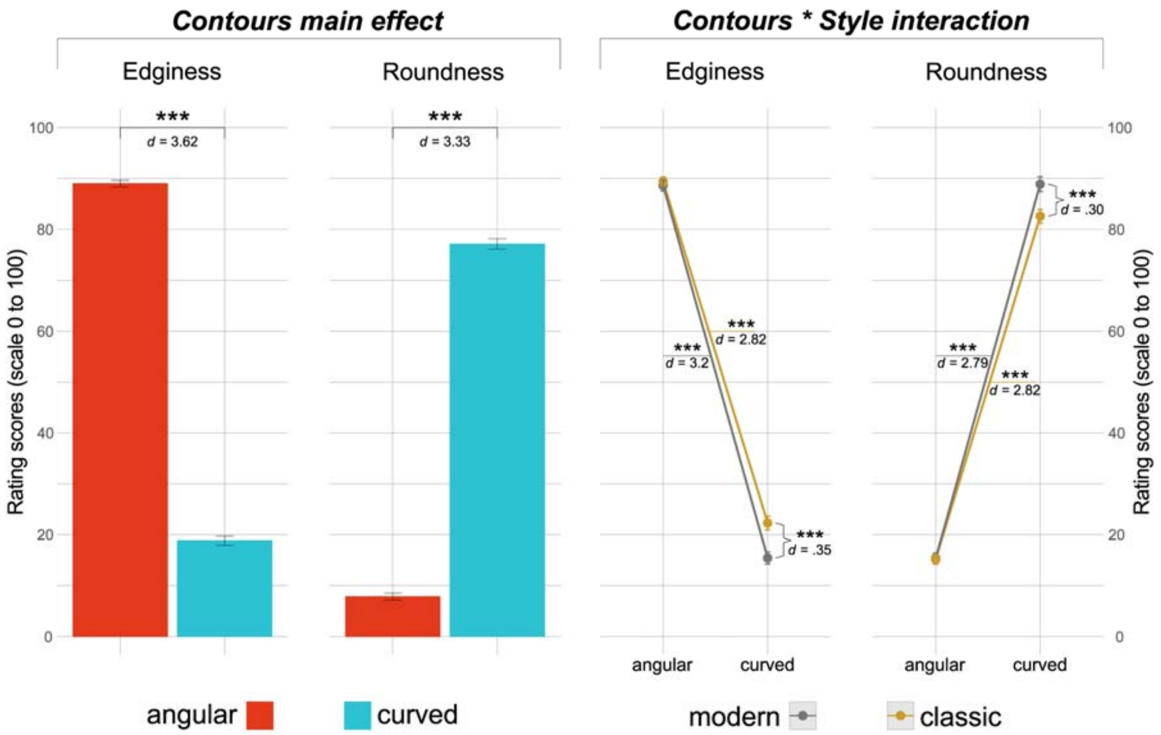

Supplementary Figure 1. Results of the manipulation check. Left: There was a main effect of contours on both *edginess* and *roundness* scores. Images of angular contours were rated significantly higher on *edginess* and lower on *roundness* than those depicting curved ones. **Right:** Interaction effect of contours with style, showing consistency in ratings of *edginess* and *roundness* within the two styles. Scoring is on a range of 0-100. Bar graphs represent mean scores; error bars indicate standard errors. Asterisks represent significance, *p < .05, **p < .01, *** p < .001.

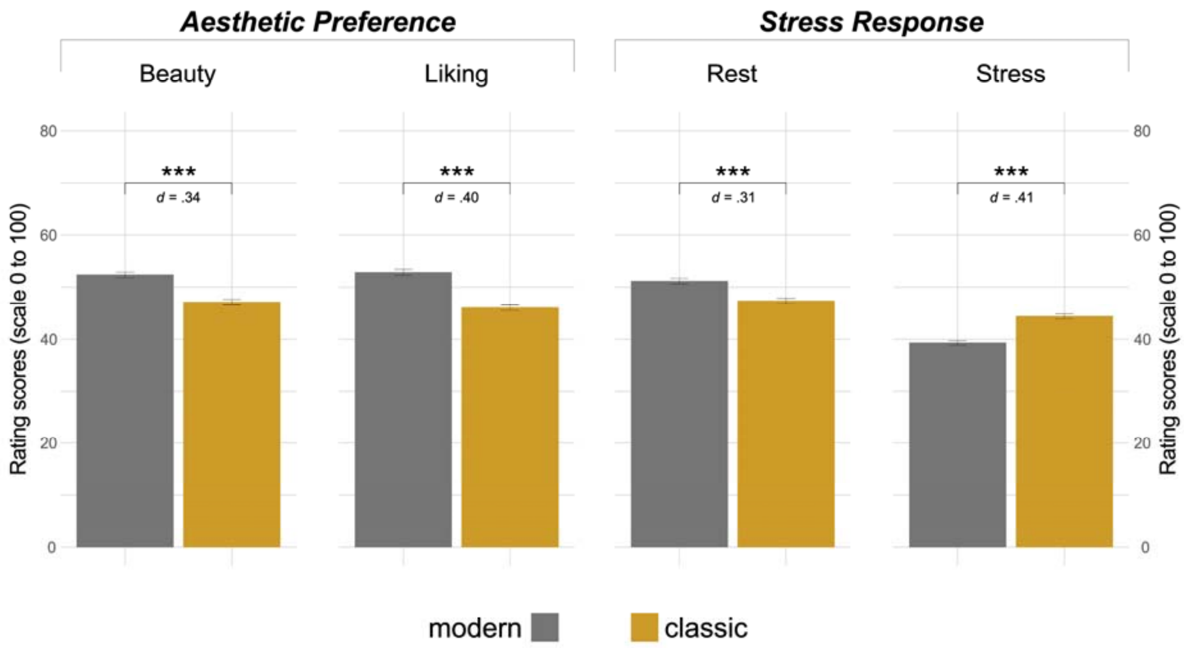

Supplementary Figure 2. Style main effect. Left to right: Results of the analyses comparing mean scores of images showing modern versus classic style on the four rating scales representing aesthetic preference (*beauty* and *liking*) and stress response (*rest* and *stress*) evaluations. Modern images were found to be more *beautiful*, more *liked*, more *restful*, and less *stressful* than classic ones. Scoring is on a range of 0-100. Bar graphs represent mean scores; error bars indicate standard errors. Asterisks represent significance, *p < .05, **p < .01, *** p < .001.

2.2 Supplementary Tables

2.2.1 Rating Tasks

Supplementary Table 1. Details of the two sets of rating tasks: set 1 (General Appraisal Scale, GAS), and set 2 (Aesthetic and Stress Response, AES) in the original German version (English translations can be found within the main manuscript).

Scale question	Anchored statements
**GAS**	
Edginess<br>"Als wie eckig empfinden Sie diesen Raum?"	0="überhaupt nicht eckig"<br>100="sehr eckig"
Roundness<br>"Als wie rund empfinden Sie diesen Raum?"	0="überhaupt nicht rund"<br>100="sehr rund"
Curiosity<br>"Wie Neugierde erweckend erscheint Ihnen dieser Raum?"	0="überhaupt nicht Neugierde erweckend"<br>100="sehr Neugierde erweckend"
Novelty<br>"Wie neuartig erscheint Ihnen dieser Raum?"	0="überhaupt nicht neuartig"<br>100="sehr neuartig’
Order/ Structure<br>"Als wie strukturiert/ geordnet empfinden Sie diesen Raum?"	0="sehr unstrukturiert/ ungeordnet"<br>100="sehr strukturiert/ geordnet"
Complexity<br>“Wie komplex erscheint Ihnen dieser Raum?"	0="überhaupt nicht komplex"<br>100="sehr komplex"
**ASR**	
Beauty<br>“Bitte schätzen Sie die Schönheit/Ästhetik des Innenraums auf dem Bild ein."	0="überhaupt nicht schön"<br>100="sehr schön"
Liking<br>"Wie gut gefällt Ihnen der Innenraum auf dem Bild?"	0="überhaupt nicht"<br>100="sehr gut"
Rest<br>“Stellen Sie sich vor Sie wären in dem Innenraum auf dem Bild. Wie erholsam wirkt der Raum auf Sie?"	0="überhaupt nicht erholsam"<br>100="sehr erholsam"
Stress/ Emotion<br>"Stellen Sie sich vor Sie wären in dem Innenraum auf dem Bild. Wie würden Sie ihre emotionale Reaktion beschreiben?"	0="entspannt"<br>100="gestresst"

2.2.2 Manipulation check

Supplementary Table 2. Results of the 2(contours) x 2(style) ANOVA for the dependent variable (rating score), shown separately for 'edginess' and 'roundness' scales.

Edginess

Predictor	df_{Num}	df_{Den}	SS_{Num}	SS_{Den}	F	p	η^2_g
(Intercept)	1	196	2287990.51	59760.74	7504.03	.000	.92
contours	1	196	969294.12	74006.13	2567.11	**.000**	.83
style	1	196	2897.36	35223.89	16.12	**.000**	.01
contours x style	1	196	1842.67	33285.58	10.85	**.001**	.01

Roundness

Predictor	df_{Num}	df_{Den}	SS_{Num}	SS_{Den}	F	p	η^2_g
(Intercept)	1	196	1422475.13	56499.87	4934.62	.000	.87
contours	1	196	944552.70	85180.30	2173.42	**.000**	.82
style	1	196	2021.12	35875.88	11.04	**.001**	.01
contours x style	1	196	1767.01	34115.99	10.15	**.002**	.01

Note. df_{Num} indicates degrees of freedom numerator. df_{Den} indicates degrees of freedom denominator. SS_{Num} indicates sum of squares numerator. SS_{Den} indicates sum of squares denominator. η^2_g indicates generalized eta-squared.

Supplementary Table 3. Descriptive statistics for dependent variable (rating score) as a function of contours, shown separately for each of the edginess and roundness scales, meant as a manipulation check of the contour contrast in the stimulus set.

	contours				post-hoc			
	angular		curved		t-test			
N= 197	*M*	*SD*	*M*	*SD*	*df*	*t*	*p*	*d*
Edginess	88.96	11.22	18.81	14.68	196	50.67	**<.0001**	3.62
Roundness	7.87	10.26	77.11	16.00	196	-46.62	**<.0001**	3.33

Note. *M* and *SD* represent mean and standard deviation, respectively. Pairwise post-hoc analysis include degrees of freedom (*df*), the size of the difference relative to the variation (t), significance (*p*-value), and effect size (*d* = cohen's d).

Supplementary Table 4. Descriptive statistics for dependent variable (rating score) as a function of style, shown separately for each of the edginess and roundness scales.

	style				post-hoc			
	modern		classic		t-test			
N=197	*M*	*SD*	*M*	*SD*	*df*	*t*	*p*	*d*
Edginess	51.97	10.88	55.80	11.13	196	-4.02	**.0001**	.29

Roundness		44.09	11.04	40.89	10.66	196	3.32	**.0011**	.24

Note. *M* and *SD* represent mean and standard deviation, respectively. Pairwise post-hoc analysis include degrees of freedom (*df*), the size of the difference relative to the variation (t), significance (*p*-value), and effect size (*d* = cohen's d).

Supplementary Table 5. Means and standard deviations for dependent variable (rating score) as a function of a 2(contours) x 2(style) design, along with the results of the post-hoc pairwise t-tests, shown separately for each of the edginess and roundness scales.

		contours				post-hoc			
		angular		curved		t-test			
N=197	style	*M*	*SD*	*M*	*SD*	*df*	*t*	*p*	*d*
Edginess	modern	88.57	15.08	15.37	16.52	196	44.75	**<.0001**	3.20
	classic	89.35	13.20	22.26	18.90	196	39.52	**<.0001**	2.82
Roundness	modern	7.97	13.42	80.21	20.01	196	-39.08	**<.0001**	2.79
	classic	7.76	13.10	74.01	18.11	196	-39.86	**<.0001**	2.82

Note. *M* and *SD* represent mean and standard deviation, respectively. Pairwise post-hoc analysis include degrees of freedom (*df*), the size of the difference relative to the variation (t), significance (*p*-value), and effect size (*d* = cohen's d).

Supplementary Table 6. Means and standard deviations for dependent variable (rating score) as a function of a 2(style) x 2(contours) design, along with the results of the post-hoc pairwise t-tests, shown separately for each of the edginess and roundness scales.

		style				post-hoc			
		modern		classic		t-test			
N=197	contours	*M*	*SD*	*M*	*SD*	*df*	*t*	*p*	*d*
Edginess	angular	88.57	15.08	89.35	13.20	196	-0.63	.53	.04
	curved	15.37	16.52	22.26	18.90	196	-4.85	**<.0001**	.35
Roundness	angular	7.97	13.42	7.76	13.10	196	0.17	.86	.01
	curved	80.21	20.01	74.01	18.11	196	4.19	**.0001**	.30

Note. *M* and *SD* represent mean and standard deviation, respectively. Pairwise post-hoc analysis include degrees of freedom (*df*), the size of the difference relative to the variation (t), significance (*p*-value), and effect size (*d* = cohen's d).

2.2.3 Effects of contours, style, and their interaction

Supplementary Table 7. Results of the 2(contours) x 2(style) ANOVA for the dependent variable (rating score), shown separately for each dimension of the ASR.

Beauty

Predictor	df_{Num}	df_{Den}	SS_{Num}	SS_{Den}	F	p	η^2_g
(Intercept)	1	197	1958717.50	115721.99	3334.43	.000	.89
contours	1	197	3119.39	60933.62	10.09	**.002**	.01
style	1	197	5405.00	46324.85	22.99	**.000**	.02
contours x style	1	197	1764.64	14028.09	24.78	**.000**	.01

Liking

Predictor	df_{Num}	df_{Den}	SS_{Num}	SS_{Den}	F	p	η^2_g
(Intercept)	1	197	1936620.24	120929.97	3154.84	.000	.88
contours	1	197	2475.45	77137.60	6.32	**.013**	.01
style	1	197	9048.40	56945.21	31.30	**.000**	.03
contours x style	1	197	3362.08	14478.45	45.75	**.000**	.01

Rest

Predictor	df_{Num}	df_{Den}	SS_{Num}	SS_{Den}	F	p	η^2_g
(Intercept)	1	197	1918760.98	107523.00	3515.49	.000	.90
contours	1	197	27357.53	54341.73	99.18	**.000**	.12
style	1	197	2843.94	29824.36	18.79	**.000**	.01
contours x style	1	197	4319.47	9981.71	85.25	**.000**	.02

Stress/ Emotion

Predictor	df_{Num}	df_{Den}	SS_{Num}	SS_{Den}	F	p	η^2_g
(Intercept)	1	197	1384315.73	80517.01	3386.99	.000	.89
contours	1	197	15438.90	47668.32	63.80	**.000**	.08
style	1	197	5221.63	30678.99	33.53	**.000**	.03
contours x style	1	197	1486.94	11767.92	24.89	**.000**	.01

Note. df_{Num} indicates degrees of freedom numerator. df_{Den} indicates degrees of freedom denominator. SS_{Num} indicates sum of squares numerator. SS_{Den} indicates sum of squares denominator. η^2_g indicates generalized eta-squared.

Supplementary Table 8. Means and standard deviations for dependent variable (rating score) as a function of contours, along with the results of the post-hoc pairwise t-tests, shown separately for each dimension of the ASR.

	contours				post-hoc			
	angular		curved		t-test			
N=198	*M*	*SD*	*M*	*SD*	*df*	*t*	*p*	*d*
Beauty	47.75	15.07	51.72	14.87	197	-3.18	**.002**	.23
Liking	47.68	16.13	51.22	15.57	197	-2.51	**.01**	.18
Rest	43.34	15.30	55.10	13.30	197	-9.96	**<.0001**	.71
Stress/ Emotion	46.22	13.62	37.39	11.83	197	7.99	**<.0001**	.57

Note. M and SD represent mean and standard deviation, respectively. Pairwise post-hoc analysis include degrees of freedom (df), the size of the difference relative to the variation (t), significance (p-value), and effect size (d = cohen's d).

Supplementary Table 9. Means and standard deviations for dependent variable (rating score) as a function of style, along with the results of the post-hoc pairwise t-tests, shown separately for each dimension of the ASR.

	style				post-hoc			
	modern		classic		t-test			
N=198	*M*	*SD*	*M*	*SD*	*df*	*t*	*p*	*d*
Beauty	52.34	13.62	47.12	15.02	197	4.79	**<.0001**	.34
Liking	52.83	14.70	46.07	15.34	197	5.60	**<.0001**	.40
Rest	51.12	12.64	47.33	13.74	197	4.33	**<.0001**	.31
Stress/ Emotion	39.24	11.02	44.38	12.68	197	-5.790	**<.0001**	.41

Note. M and SD represent mean and standard deviation, respectively. Pairwise post-hoc analysis include degrees of freedom (df), the size of the difference relative to the variation (t), significance (p-value), and effect size (d = cohen's d).

Supplementary Table 10. Means and standard deviations for dependent variable (rating score) as a function of a 2(contours) x 2(style) design, along with the results of the post-hoc pairwise t-tests, shown separately for each dimension of the ASR.

		contours				post-hoc			
		angular		curved		t-test			
N=198	style	*M*	*SD*	*M*	*SD*	*df*	*t*	*p*	*d*
Beauty	modern	48.87	17.01	55.82	16.06	197	-5.21	**<.0001**	.37
	classic	46.63	16.87	47.61	19.27	197	-0.68	0.49	.05
Liking	modern	49.00	19.32	56.66	16.87	197	-5.07	**<.0001**	.36

	classic	46.36	17.46	45.78	20.13	197	0.38	0.71	.03
Rest	modern	42.90	16.76	59.33	14.07	197	-12.95	**<.0001**	.92
	classic	43.78	16.04	50.87	16.95	197	-5.45	**<.0001**	.39
Stress/	modern	45.03	15.13	33.45	12.77	197	9.43	**<.0001**	.67
Emotion	classic	47.42	14.90	41.33	15.88	197	4.90	**<.0001**	.35

Note. M and SD represent mean and standard deviation, respectively. Pairwise post-hoc analysis include degrees of freedom (df), the size of the difference relative to the variation (t), significance (p-value, corrected using the "FDR" method), and effect size (d = cohen's d).

Supplementary Table 11. Means and standard deviations for dependent variable (rating score) as a function of a 2(style) x 2(contour) design, along with the results of the post-hoc pairwise t-tests, shown separately for each dimension of the ASR.

		style				post-hoc			
		modern		classic		t-test			
N=990	style	*M*	*SD*	*M*	*SD*	*df*	*t*	*p*	*d*
Beauty	angular	48.87	17.01	46.63	16.87	197	2.04	**.057**	.15
	curved	55.82	16.06	47.61	19.27	197	5.98	**<.0001**	.43
Liking	angular	49.00	19.32	46.36	17.46	197	2.09	**.05**	.15
	curved	56.66	16.87	45.78	20.13	197	7.57	**<.0001**	.54
Rest	angular	42.90	16.76	43.78	16.04	197	-1.04	0.30	.07
	curved	59.33	14.07	50.87	16.95	197	7.34	**<.0001**	.52
Stress/	angular	45.03	15.13	47.42	14.90	197	-2.66	**0.008**	.19
Emotion	curved	33.45	12.77	41.33	15.88	197	-6.73	**<.0001**	.48

Note. M and SD represent mean and standard deviation, respectively. Pairwise post-hoc analysis include degrees of freedom (df), the size of the difference relative to the variation (t), significance (p-value, corrected using the "FDR" method), and effect size (d = cohen's d).

2.2.4 Results of the two-way interaction of contours*sex

Supplementary Table 12. Results of the mixed ANOVA with 2(contours) as within-subject factors and 2(sex) as a between-subject factor performed on the dependent variable (rating score), and shown separately for each dimension of the ASR.

Beauty

Predictor	df_{Num}	df_{Den}	SS_{Num}	SS_{Den}	F	p	η^2_g
(Intercept)	1	196	979358.75	57848.01	3318.25	.000	.92
sex	1	196	12.98	57848.01	0.04	.834	.00
contours	1	196	1559.69	28949.69	10.56	.001	.02
**sex x contours**	**1**	**196**	**1517.12**	**28949.69**	**10.27**	**.002**	**.02**

Liking

Predictor	df_{Num}	df_{Den}	SS_{Num}	SS_{Den}	F	p	η^2_g
(Intercept)	1	196	968310.12	60464.44	3138.85	.000	.91
sex	1	196	0.55	60464.44	0.00	.966	.00
contours	1	196	1237.73	36930.34	6.57	.011	.01
**sex x contours**	**1**	**196**	**1638.46**	**36930.34**	**8.70**	**.004**	**.02**

Rest

Predictor	df_{Num}	df_{Den}	SS_{Num}	SS_{Den}	F	p	η^2_g
(Intercept)	1	196	959380.49	53686.97	3502.50	.000	.92
sex	1	196	74.53	53686.97	0.27	.603	.00
contours	1	196	13678.76	25702.28	104.31	.000	.15
**sex x contours**	**1**	**196**	**1468.58**	**25702.28**	**11.20**	**.001**	**.02**

Stress

Predictor	df_{Num}	df_{Den}	SS_{Num}	SS_{Den}	F	p	η^2_g
(Intercept)	1	196	692157.86	39986.17	3392.75	.000	.92
sex	1	196	272.34	39986.17	1.33	.249	.00
contours	1	196	7719.45	23118.91	65.44	.000	.11
**sex x contours**	**1**	**196**	**715.24**	**23118.91**	**6.06**	**.015**	**.01**

Note. df_{Num} indicates degrees of freedom numerator. df_{Den} indicates degrees of freedom denominator. SS_{Num} indicates sum of squares numerator. SS_{Den} indicates sum of squares denominator. η^2_g indicates generalized eta-squared.

Supplementary Table 13. Means and standard deviations for dependent variable (rating score) as a function of a 2(contours) x 2(sex) design, along with the results of the post-hoc pairwise t-tests, shown separately for each dimension of the ASR.

		contours				post-hoc			
		angular		curved		t-test			
N=99	sex	*M*	*SD*	*M*	*SD*	*df*	*t*	*p*	*d*
Beauty	male	49.52	15.74	49.58	16.23	196	-0.03	.97	.002
	female	45.97	14.23	53.85	13.11	196	-4.56	**<.0001**	.33
Liking	male	49.75	17.48	49.22	16.76	196	0.27	.79	0.02
	female	45.61	14.45	53.21	14.09	196	-3.90	**.0005**	.28
Rest	male	45.70	15.97	53.61	15.07	196	-4.86	**<.0001**	.35
	female	40.98	14.28	56.59	11.13	196	-9.59	**<.0001**	.68
Stress/	male	44.05	14.45	37.91	12.89	196	3.98	**.0002**	.28
Emotion	female	48.40	12.41	36.88	10.70	196	7.46	**<.0001**	.53

Note. M and SD represent mean and standard deviation, respectively. Pairwise post-hoc analysis include degrees of freedom (df), the size of the difference relative to the variation (t), significance (p-value, corrected using the "FDR" method), and effect size (d = cohen's d). The terms 'male' and 'female' are used as grouping adjectives, as this was how participants were asked to (dichotomously) classify themselves.

Supplementary Table 14. Means and standard deviations for dependent variable (rating score) as a function of a 2(sex) x 2(contours) design, along with the results of the post-hoc pairwise t-tests, shown separately for each dimension of the ASR.

		sex				post-hoc			
		male		female		t-test			
N=99	sex	*M*	*SD*	*M*	*SD*	*df*	*t*	*p*	*d*
Beauty	angular	49.52	15.74	45.97	14.23	196	1.67	.13	.12
	curved	49.58	16.23	53.85	13.11	196	-2.04	.09	.15
Liking	angular	49.75	17.48	45.61	14.45	196	1.82	.09	.13
	curved	49.22	16.76	53.21	14.09	196	-1.82	.09	.13
Rest	angular	45.70	15.97	40.98	14.28	196	2.19	**.04**	.16
	curved	53.61	15.07	56.59	11.13	196	-1.59	.11	.11
Stress/	angular	44.05	14.45	48.40	12.41	196	-2.27	**.03**	.16
Emotion	curved	37.91	12.89	36.88	10.70	196	0.61	.54	.04

Note. M and SD represent mean and standard deviation, respectively. Pairwise post-hoc analysis include degrees of freedom (df), the size of the difference relative to the variation (t), significance (p-value, corrected using the "FDR" method), and effect size (d = cohen's d). The terms 'male' and 'female' are used as grouping adjectives, as this was how participants were asked to (dichotomously) classify themselves.

Supplementary Table 15. Correlation coefficients computed in R using the function 'rmcorr'. Following the guidelines provided in Bakdash and Marusich (2017), data was stored in long format with separate columns for participant and each of the four measures scores, and separate rows for each observation labeled by participant (N=198 with 3,960 observations in total). The function handles repeated measures data without violating independence assumptions or requiring first averaging the data. Paired correlations were computed separately for each of the possible pairs of the rating dimensions, and are reported in the matrix below.

**N=198 participants**<br>**N=3,960 observation**	***Beauty***	***Liking***	***Rest***	***Stress***
Beauty	1			
Liking	0.78	1		
Rest	0.69	0.70	1	
Stress	−0.55	−0.57	−.58	1
